# Europium Doped Atomically Precise Bismuth Oxido Nanoclusters as Molecular Building Blocks for Photoluminescent Hybrid Materials

**DOI:** 10.1002/chem.71580

**Published:** 2026-08-17

**Authors:** Rico Thomas, Senthil Kumar Kuppusamy, Tobias Rüffer, Marcus Weber, Mario Ruben, Michael Mehring

**Affiliations:** ^1^ Faculty of Natural Sciences, Institute of Chemistry, Coordination Chemistry Chemnitz University of Technology Chemnitz Germany; ^2^ Karlsruhe Institute of Technology (KIT), Institute for Quantum Materials and Technologies (IQMT) Karlsruhe Germany; ^3^ Faculty of Natural Sciences Institute of Chemistry, Inorganic Chemistry Chemnitz University of Technology Chemnitz Germany; ^4^ Center of Materials Architectures and Integration of Nanomembranes Chemnitz University of Technology Chemnitz Germany; ^5^ Karlsruhe Institute of Technology (KIT) Institute of Nanotechnology (INT) Karlsruhe Germany; ^6^ Centre Européen de Sciences Quantiques (CESQ) Institut De Science et D'ingénierie Supramoléculaires (ISIS) Strasbourg France

**Keywords:** bismuth, cluster compounds, doping, fluorescence, lanthanides

## Abstract

Structural and optical characterization of Eu^3+^ doped atomically precise bismuth oxido nanoclusters (BiO‐NCs) of about 2 nm in size is reported. The BiO‐NC [Bi_38_O_45_(NO_3_)_24_(dmso)_28_]:Eu (**C‐1_D_:Eu**) is transformed into soluble methacrylate (^−^OMc) coordinated [Bi_38_O_45_(OMc)_24_(dmso)_4_]:Eu·4dmso·4H_2_O (**C‐2_D_:Eu**) and [Bi_38_O_45_(OMc)_24_(EtOH)_10_]:Eu·4EtOH (**C‐2_E_:Eu**) by ligand exchange and without changing the amount of Eu^3+^ doping (*ω* ≈ 1%). Based on SC XRD analysis of **C‐1_D_:Eu** and **C‐2_E_:Eu** partial substitution of the bismuth atoms with preference in the inner {Bi_6_O_9_} core of the {Bi_38_O_45_} cluster is suggested. Temperature dependent photoluminescence studies show bismuth‐ and europium‐based dual emission at low temperatures, whereas only the latter is preserved at room temperature. As a result of the low‐symmetry environment of Eu^3+^ a remarkable intensity of the ^5^D_0_→^7^F_0_ (electric dipole) transition is observed, accompanied by lifetimes of about 2 ms and quantum yields ranging from 53% (**C‐2_D_:Eu)** to 65 % (**C‐1_D_:Eu)**. The solubility of **C‐2_E_:Eu** enables its use for the synthesis of organic–inorganic hybrid materials. Thus, transparent hybrid materials were prepared by radical copolymerization of methyl methacrylate, 2‐hydroxyethyl methacrylate and **C‐2_E_:Eu**, showing distinct photoluminescence characteristics even with a low Eu^3+^ content of 2.7·10^−5^ mol%.

## Graphical abstract

Europium doping of bismuth oxido nanoclusters (BiO‐NCs) provides stable hosts for Eu^3+^ in an asymmetric coordination environment, which in turn results in an intense ^5^D_0_→^7^F_0_ transition and long luminescence lifetimes. Transparent hybrid materials showing characteristic red photoluminescence are accessible via radical copolymerisation of these soluble nanoclusters

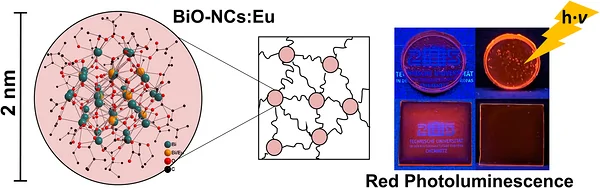

## 1 Introduction

Interest in lanthanide oxido clusters has grown over the past decades due to their structural diversity and versatile magnetic and luminescent properties [1, 2, 3]. In addition to Sm^3+^, Tb^3+^, and Dy^3+^, the focus is mainly on Eu^3+^ for luminescence studies due to its outstanding spectral properties. As result of highly intense red luminescence with a long lifetime it is used in LEDs, fluorescent lamps or anti‐counterfeiting markers as used in security labels among others [4, 5, 6, 7, 8, 9]. A key feature is the structural environment of Eu^3+^, such as coordination number and geometry, which strongly determines the luminescence properties. Noteworthy, the luminescence fingerprint of europium might also be used as spectroscopic probe for site symmetry estimation in solid state materials [10, 11]. Recently, it has been demonstrated that the alluring optical properties of molecular Eu^3+^ complexes such as ultranarrow spectral linewidths associated with the ^5^D_0_→^7^F_0_ transition could be leveraged to construct coherent light‐molecule interfaces, useful for quantum information processing applications (QIP) [12, 13, 14]. For QIP application, the intensity and width of the induced electric dipole ^5^D_0_→^7^F_0_ transition, as typically observed in compounds of low symmetry sites of europium plays a key role [10]. In addition, diverse materials such as Eu^3+^ doped ceramics [15], glasses [16], liquid crystals [17], organic–inorganic hybrid materials [18, 19], and nanostructured materials [11, 20] have been investigated and might provide a coordination environment of low symmetry for europium [10, 21], but often suffer from a poorly defined chemical environment of the dopant. Recently, we have demonstrated, that bismuth in nanoclusters (BiO‐NCs) with a {Bi_38_O_45_} core structure can be partially substituted with lanthanides, whereby the bismuth oxido core provides a matrix for the lanthanide in a low‐symmetric coordination environment [22, 23, 24]. Due to the similarity between bismuth and europium with regard to size and coordination geometries it seemed promising to have closer look to europium to generate light emitters with narrow line‐widths. Noteworthy, heterobimetallic bismuth‐based MO‐NCs are rare and only a few have been reported incorporating lanthanides [22, 23, 24, 25, 26], alkali metals [27, 28, 29] and titanium [30, 31]. However, interest in smaller, mainly hexanuclear, metal oxido clusters containing lanthanides with focus on hydrolysis processes and properties is of ongoing interest [32, 33, 34, 35, 36, 37, 38].

Herein, we report on the synthesis, structural characterization [23] and photoluminescence properties of europium doped BiO‐NCs including [Bi_38_O_45_(NO_3_)_24_(dmso)_28_]:Eu (**C‐1_D_:Eu**) as well as soluble methacrylate (^−^OMc) coordinated [Bi_38_O_45_(OMc)_24_(dmso)_4_]:Eu·4dmso·4H_2_O (**C‐2_D_:Eu**) and [Bi_38_O_45_(OMc)_24_(EtOH)_10_]:Eu·4EtOH (**C‐2_E_:Eu**). The latter is demonstrated to be a suitable molecular precursor for optical transparent and photoluminescent organic–inorganic hybrid materials.

## 2 Results and Discussion

### 2.1 Synthesis and Structural Characterization of Europium Doped BiO‐NCs

The europium doped BiO‐NC [Bi_38_O_45_(NO_3_)_24_(dmso)_28_]:Eu (**C‐1_D_:Eu**) was synthesized by simultaneous alkaline‐induced hydrolysis and condensation of Bi(NO_3_)_3_·5H_2_O and Eu(NO_3_)_3_·6H_2_O in dmso, followed by subsequent crystallization via acetone vapor diffusion into the reaction mixture under ambient conditions. A ligand exchange reaction using sodium methacrylate (NaOMc) to give the dmso solvate [Bi_38_O_45_(OMc)_24_(dmso)_4_]:Eu·4dmso·4H_2_O (**C‐2_D_:Eu**) was performed. Finally, the coordinated dmso was replaced by crystallization from EtOH to give the ethanol solvate [Bi_38_O_45_(OMc)_24_(EtOH)_10_]:Eu·4EtOH (**C‐2_E_:Eu,** sample 1), which was converted into solvent‐free [Bi_38_O_45_(OMc)_24_]:Eu (**C‐2:Eu**) upon gentle heating in vacuum. Recrystallisation of the ethanol solvate **C‐2_E_:Eu** from DMSO reproduced single crystals of the dmso solvate [Bi_38_O_45_(OMc)_24_(dmso)_4_]:Eu·4dmso·4H_2_O **(C‐2_E_:Eu,** sample 2) (Scheme 1).

**SCHEME 1:**
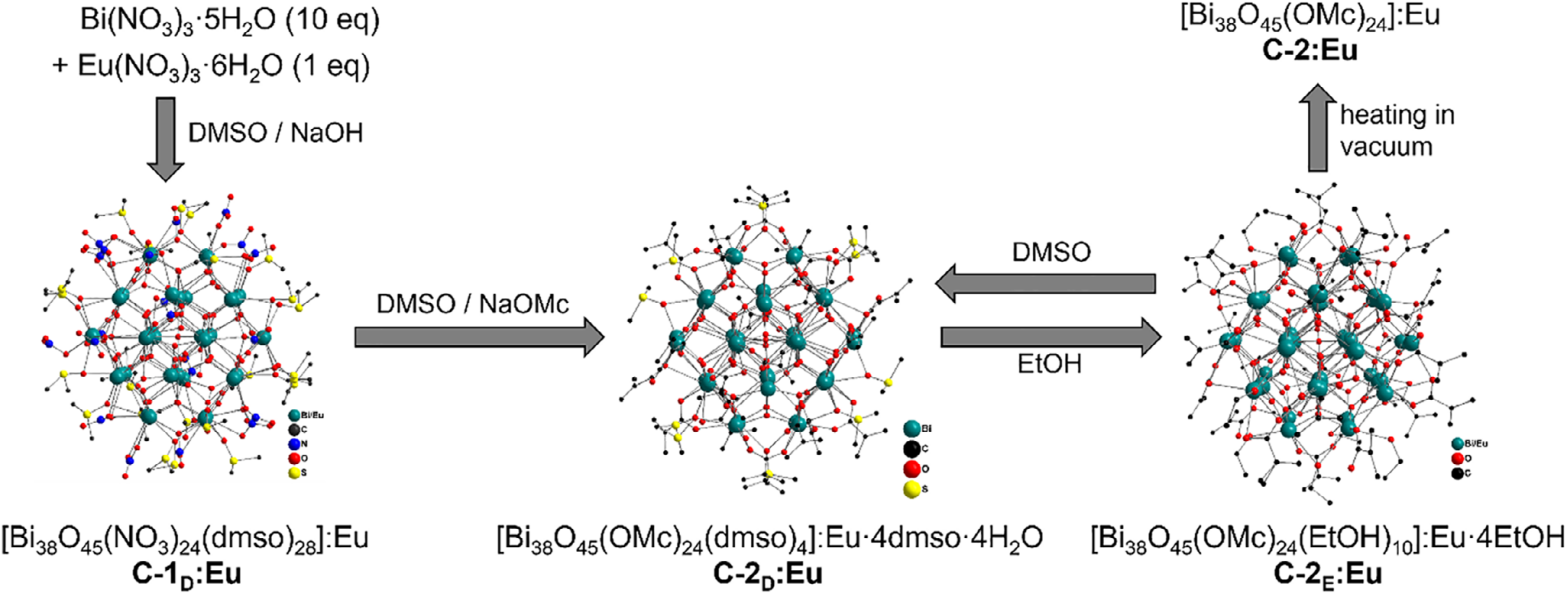
Synthetic pathway to produce europium doped BiO‐NCs.

Quantification of the europium content using inductive coupled plasma optical emission spectroscopy (ICP‐OES) for the as‐prepared compounds **C‐1_D_:Eu**—**C‐2_E_:Eu** was carried out (Table S1), showing europium contents of about 1 ω%. In combination with elemental analysis compositions were determined providing the formulas [Bi_37.17_Eu_0.83_O_45_(NO_3_)_24_(dmso)_26_] (**C‐1_D_:Eu**, *ω*(Eu) = 1.02 ω%), [Bi_37.34_Eu_0.66_O_45_(OMc)_24_(dmso)_6_] (**C‐2_D_:Eu**, *ω*(Eu) = 0.88 ω%), and [Bi_37.32_Eu_0.68_O_45_(OMc)_24_] (**C‐2:Eu**, *ω*(Eu) = 0.97 ω%). The non‐stochiometric cluster core composition of the Eu^3+^ doped BiO‐NCs indicates non‐stoichiometric incorporation of Eu^3+^ leading to a mixture of undoped and doped BiO‐NCs in the bulk material.

In order to proof the chemical composition and stability of the here reported Eu^3+^ doped BiO‐NCs ESI‐MS spectra were recorded (Figure S3–Figure S11; Tables S2 and S3). Applying soft ESI conditions results in the generation of doubly, triply, and quadruply positive charged cations from BiO‐NC **C‐1_D_:Eu**, while preserving the majority of the respective ligands and some dmso. The ionization behavior of BiO‐NC **C‐1_D_:Eu** (Figure S4), with regard to the loss of nitrate anions and dmso is almost identical to the undoped BiO‐NC **C‐1_D_**. [22, 39] The 4+ charge state is dominated by [Bi_38_O_46_(NO_3_)_18_(dmso)*_y_*]^4+^ (*y* = 7, **II**, *m*/*z* = 2584.7243; *y* = 6, **III**, *m*/*z* = 2565.2208). However, species with increasing dopant content are clearly assigned starting from [Bi_37_EuO_46_(NO_3_)_18_(dmso)_6_]^4+^ (**V**, *m*/*z* = 2551.2061) up to [Bi_34_Eu_4_O_46_(NO_3_)_18_(dmso)_6_]^4+^ (**VIII**, *m*/*z* = 2508.6611) (Table S2). In case of the methacrylate coordinated BiO‐NCs **C‐2_D_:Eu** and **C‐2_E_:Eu**, the formation of doubly and triply positive charged BiO‐NCs is preferred. Other than that, the results of the cluster core compositions are similar to those of **C‐1_D_:Eu**. A cut out of the survey ESI mass spectrum of **C‐2_E_:Eu** (Figure 1) is provided, showing doubly positive charged species, including the calculated isotopic pattern of high intensity for [Bi_38_O_45_(OMc)_22_]^2+^ (**IX**, *m*/*z* = 5265.8311) and [Bi_37_EuO_45_(OMc)_22_]^2+^ (**XI**, *m/z* = 5237.8015). Similar to the findings on compound **C‐1_D_:Eu**, europium doped cluster cores were detected in the range from [Bi_37_EuO_45_(OMc)_22_]^2+^ up to [Bi_34_Eu_4_O_45_(OMc)_22_]^2+^ for both compounds **C‐2_D_:Eu** and **C‐2_E_:Eu**. The europium doped BiO‐NCs are best described as a mixture of undoped clusters with a homometallic {Bi_38_O_45_} cluster core and doped clusters with {Bi_38–_ *_x_* Eu*_x_* O_45_} (*x* = 1–4) motifs. The intensity ratio of signals as assigned to species with different amount of dopant is similar for **C‐1_D_:Eu, C‐2_E_:Eu,** and **C‐2_E_:Eu**. Thus, the stability and the chemical integrity of all three Eu doped BiO‐NCs was proven using ESI‐MS.

**FIGURE 1:**
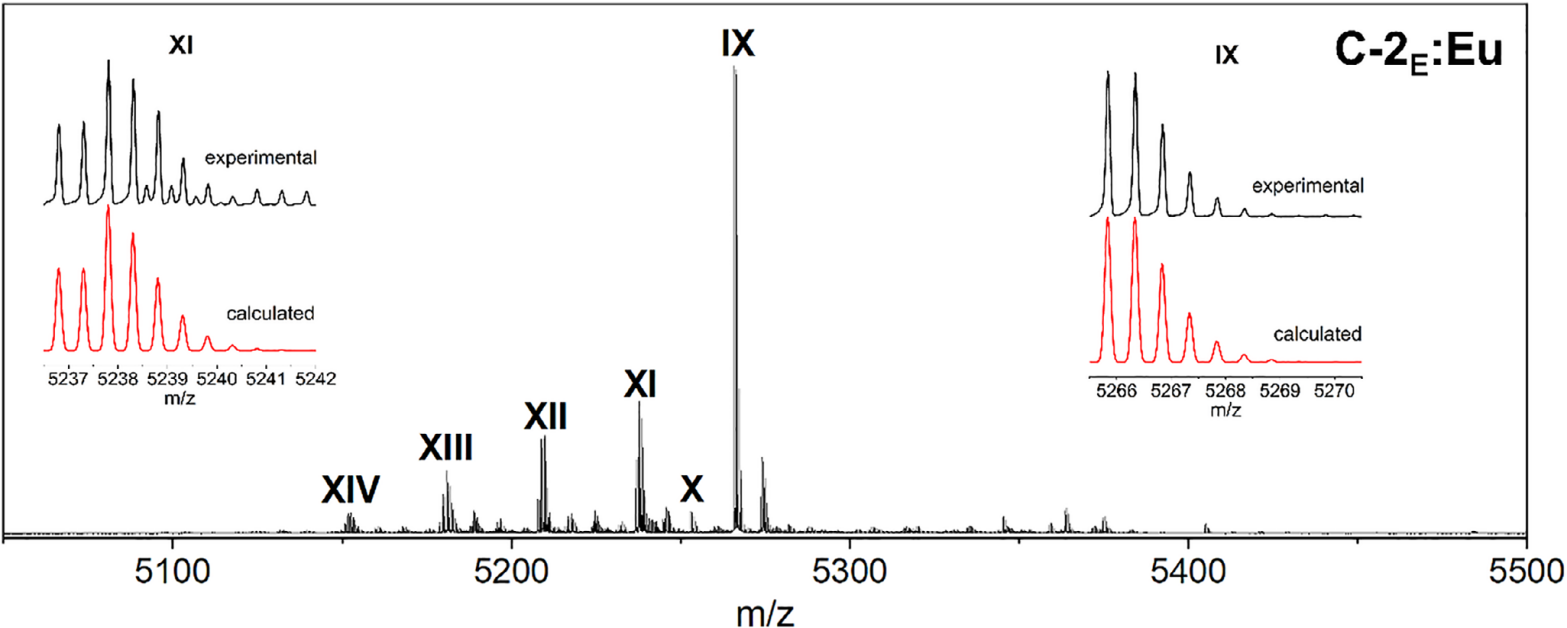
Part of the ESI mass spectrum of compound **C‐2_E_:Eu** electrosprayed from *^i^* PrOH showing doubly positive charged homometallic ([Bi_38_O_45_(OMc)_22_]^2+^ (IX), [Bi_38_O_45_(OMc)_21_(O*i*Pr)]^2+^ (X)), and heterobimetallic ([Bi_37_EuO_45_(OMc)_22_]^2+^ (XI), [Bi_36_Eu_2_O_45_(OMc)_22_]^2+^ (XII), [Bi_35_Eu_3_O_45_(OMc)_22_]^2+^ (XIII), [Bi_34_Eu_4_O_45_(OMc)_22_]^2+^ (XIV)) BiO‐NC cations.

To get closer insights, the molecular structures of the BiO‐NCs **C‐1_D_:Eu** (Figure 2), **C‐2_D_:Eu** (Figure S12), and **C‐2_E_:Eu** (Figure 2; Figures S18‐21) were determined from single crystal x‐ray diffraction (SC XRD) studies. The resulting crystallographic data sets are summarized together with the isostructural undoped host structures in the SI (Tables S4, S8, and S12). Please note that two independent SC XRD measurements were performed on crystals from different crystallization approaches of **C‐2_D_:Eu** and **C‐2_E_:Eu**.

**FIGURE 2:**
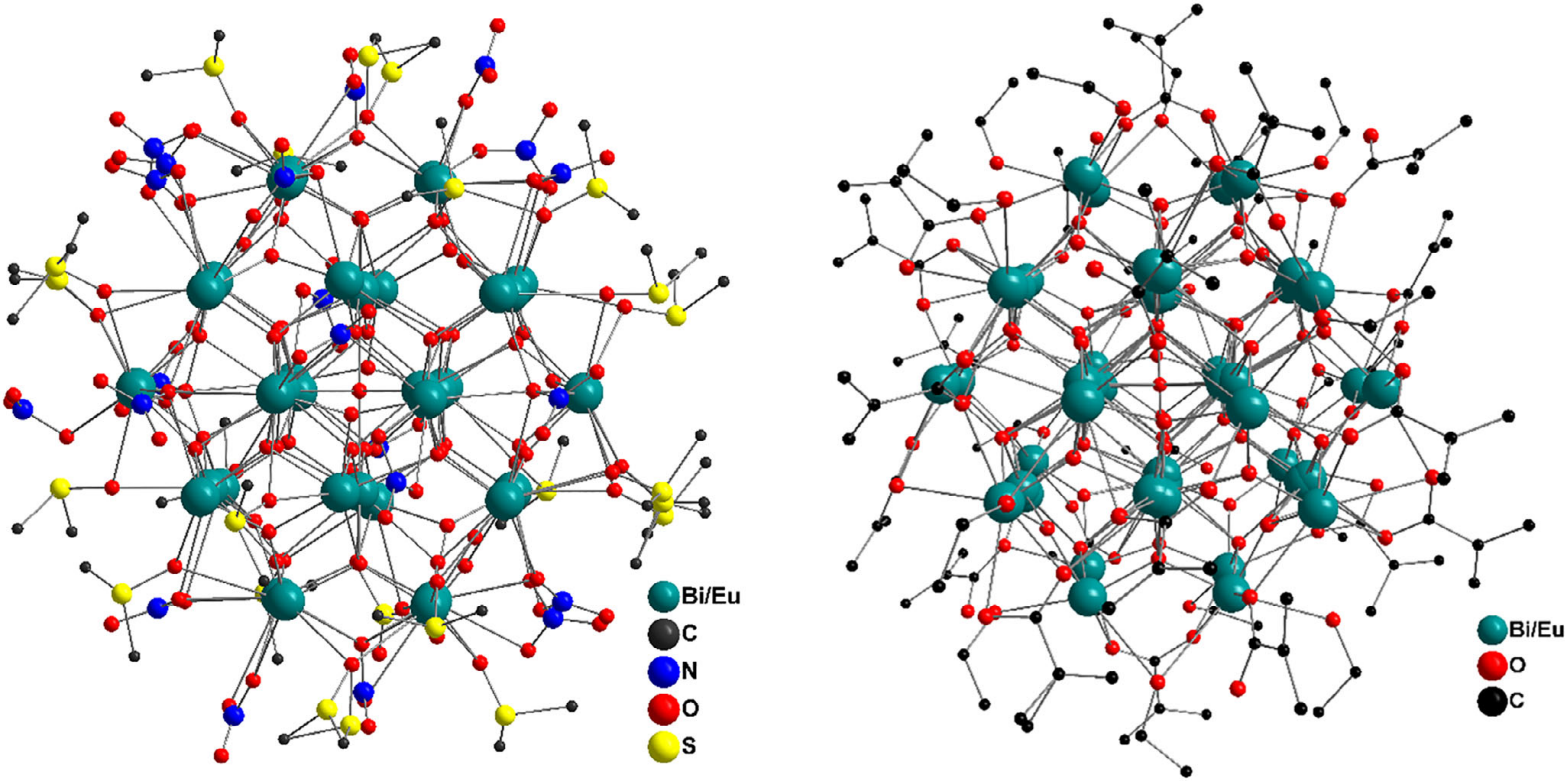
“Ball and Stick” models of the europium doped BiO‐NC **C‐1_D_:Eu** and **C‐2_E_:Eu**. Hydrogen atoms as well as non‐coordinated molecules are omitted for clarity.

The incorporation of Eu^3+^ dopants in the BiO‐NCs was determined through the refinement and comparison with the isostructural undoped host structures, and was further analyzed for the significance of Eu^3+^ doping in the respective structures in accordance to a recently published strategy taking into account the undoped host structure (details Supporting Information) [22, 23, 24]. Based on the results for the nitrate decorated cluster **C‐1_D_:Eu** it is concluded that the Eu^3+^ cations primarily occupy the inner {Bi_6_O_9_} core motif. The occupancy with Eu^3+^ decreased strongly toward the outer bismuth positions in the BiO‐NCs. This is in line with the results from electrospray‐ionization mass spectrometry revealing a mixture of species having core structures of the type {Bi_38–_ *_x_* Eu*_x_* O_45_} (*x* = 0–4). However, quantification by SC‐XRD gave increased values compared to the doping level as determined using ICP‐OES (Table S17). This is attributed to crystal and measurement specific uncertainties, discussed in detail in the Supporting Information. The data set for the methacrylate substituted cluster **C‐2_D_:Eu** would allow for the conclusion that all bismuth positions are partially occupied by europium at least to a small amount. However, occupation of all positions seemed to be unlikely because (i) the nitrate bismuth oxido clusters reported here and elsewhere indicate preference for the inner core positions [24] and (ii) former studies demonstrated the stability of the {Bi_38_O_45_} cluster core upon substitution reactions [40]. Thus, an ethanol solvate **C2_E_:Eu** (sample 1) was crystallized starting from the dmso solvate **C2_D_:Eu**. unambiguously showing preference of the inner core positions similar to the nitrate cluster **C1_D_:Eu**. This was repeated for another batch of crystals and gave a similar result. Then, these crystals were crystallized from dmso to give again crystals of the dmso solvate **C2_D_:Eu** (sample 2). The crystal structure analysis gave the same result as obtained for sample 1, again indicating partial substitution of all positions. Thus, we applied our described method of “formaly” substracting the pristine (non‐doped) structure to check the reliability of the doping positions (see detailed comment above and SI). In conclusion, reliability for the assignment of the europium positions is given for **C1_D_:Eu** and **C2_E_:Eu**, but not for **C2_D_:Eu**. However, ICP‐OES and ESI‐MS clearly verify the Eu^3+^ doping in all BiO‐NCs, and we can exclude the disassembly and reassembly of the BiO‐NCs core under the conditions applied according to previous in situ experiments [39, 40].

The Eu^3+^ doped and undoped structures show only minor differences in M─O bond lengths and coordination modes for Bi^3+^/Eu^3+^ (Tables S5, S9, and S13). As typical for bismuth in BiO‐NCs its coordination, and therefor that of the Eu^3+^ dopants as well, is asymmetric, showing strong variation in the typical M–O bond length range between 2.0–3.15 Å. Additional longer distances, but below the sum of the van der Waals radii, suggest the presence of additional M…O interactions (Tables S7 and S15). Some typical asymmetric coordination geometries are given in the SI (Figures S15 and S22). This asymmetric coordination environment for Eu^3+^ is in accordance with the emission profiles of the photoluminescence studies as discussed below.

The BiO‐NCs in solid state show a nearly hexagonal close packing, as exemplarily shown for BiO‐NC **C‐2_D_:Eu** (Figure S18). The close packed BiO‐NCs in the solid‐state structures show an averaged distance from center to center of neighboring clusters of 2.2 nm (**C‐1_D_:Eu**) and 1.9 nm (**C‐2_D_:Eu**, **C‐2_E_:Eu**), corresponding approximately to the diameter of the cluster. The powder x‐ray diffraction (PXRD) data for all doped BiO‐NCs show a good agreement with the simulated patterns generated from the SC XRD data (Figure S24).

Summing up the analysis with regard to the dopant positions, we conclude that the europium atoms are statistically distributed showing prevalence for the inner core positions. In a previous study on the cluster growth using in situ pair distribution function (PDF) and small angle x‐ray scatting (SAXS) [40], we postulated a fast growth of the {Bi_6_O_9_} core and slower growth toward the final {Bi_38_O_45_} cluster. Thus, in a first step of hydrolysis the rare earth cation seems to be trapped in the hexanuclear pre‐cluster whereas in later steps incorporation is less favorable most likely as result of slower dynamic coordination processes.

### 2.2 Optical Characterization of Europium Doped and Undoped BiO‐NCs

The optical properties of the europium doped BiO‐NCs differ markedly in comparison to the undoped BiO‐NCs. Their diffuse reflection UV–vis spectra (DRS, Figure 3) show the typical absorption characteristics of undoped BiO‐NCs with several additional transitions of low intensity, which are characteristic for Eu^3+^‐ions. The most dominant absorption bands for the europium dopant are attributed to the ^7^F_0_ → ^5^L_6_ (395 nm), ^7^F_1_ → ^5^L_6_ (402 nm), and ^7^F_0_ → ^5^D_2_ (465 nm) transitions (Table S18) [10]. The broad absorption in the UV region results from the absorption of the bismuth oxido cluster core. A bathochromic shift in the onset of this broad absorption **C‐1_D_:Eu** in comparison to **C‐2_D_:Eu** and **C‐2:Eu** is noticed, which is most likely a result of the ligand–to–metal charge‐transfer (LMCT) from the carboxylate ligands to bismuth [41, 42].

**FIGURE 3:**
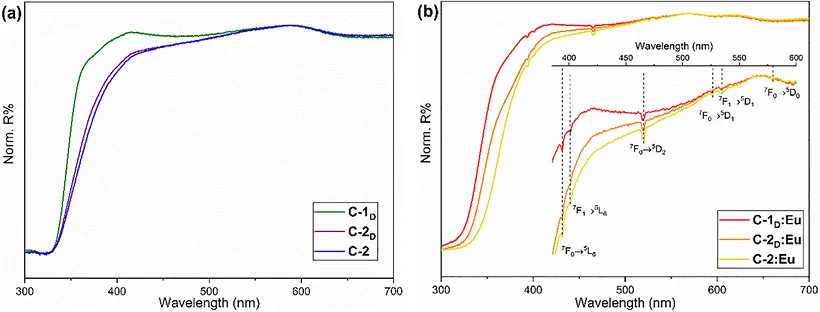
UV–vis diffuse reflectance spectra (DRS) of (a) the different undoped BiO‐NCs and (b) the europium doped BiO‐NCs, including the enlarged part between 385 and 600 nm with the assigned Eu^3+^ transitions.

### 2.3 Temperature‐Dependent Photophysical Studies of Undoped BiO‐NCs

Temperature‐dependent photoluminescent (PL) studies of undoped BiO‐NCs **C‐1_D_** and **C‐2** revealed the presence of a broad emission in the visible light region upon excitation using UV light (Figure 4). On a comparative scale, a hypsochromic shifted emission maximum is observed for nitrate‐decorated **C‐1_D_** (*λ*_max_ *_._* = 540 nm) relative to methacrylate‐decorated **C‐2** (*λ*_max_ *_._* = 601 nm). The emission intensity decreased with increasing temperature, and at 300 K no discernible emission was observed for the BiO‐NCs. The emission colors of the undoped BiO‐NCs from the emission profiles acquired at 2.4 K were determined with Commission Internationale de l'Eclairage (CIE) 1931 coordinates (Figure S25) and are best described as yellow–green (**C‐1_D_**) and yellow–orange (**C‐2**). Photoluminescence excitation (PLE) studies of **C‐1_D_** by monitoring emission at 540 nm revealed two distinct transitions, a relatively intense transition centered at 314 nm (31847 cm^−1^) and a weak shoulder‐like one at 338 nm (29586 cm^−1^). Exciting **C‐1_D_** at 314 and 334 nm led to the observation of comparable emission profiles at 2.4 K (Figure S 26). The intensity variation in the emission profiles is attributed to the variation of optical densities at the excitation wavelengths. For **C‐2**, PLE profiles with maxima centered at 314 nm have been observed (Figure 4b). The excitation bands observed for **C‐1_D_** and **C‐2** at 314 nm (Figure 4 a,b) can be attributed either to the direct ^3^P_1_ ← ^1^S_0_ transition of Bi^3+^ or to a ligand‐to‐metal charge‐transfer (LMCT) band form O^2–^ to Bi^3+^ [43].

**FIGURE 4:**
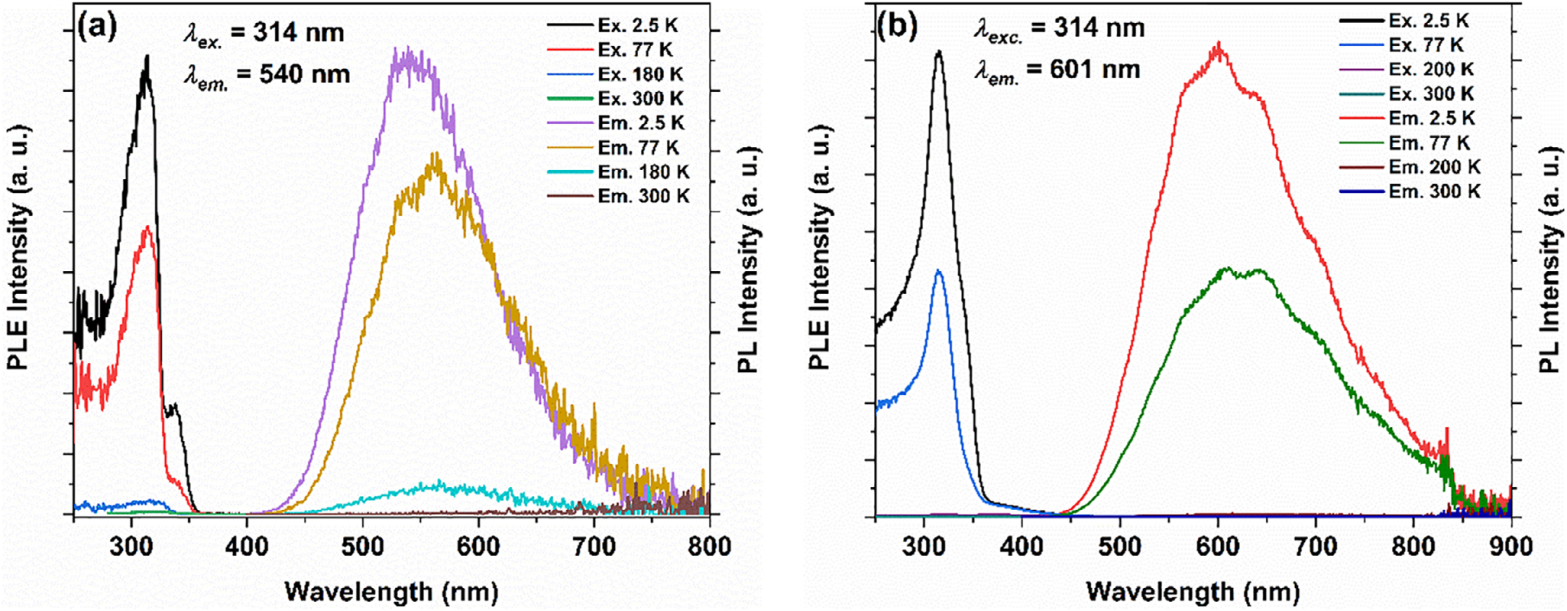
(a) Temperature‐dependent PLE and PL studies of undoped BiO‐NC C‐1_D_, (b) Temperature‐dependent PLE and PL studies of undoped BiO‐NC C‐2.

Microsecond long lifetimes—*τ*_1_ = 65 µs and *τ*_2_ = 143 µs (**C‐1_D_**, *λ*_em._ = 540 nm) and *τ*_1_ = 38 µs and *τ*_2_ = 110 µs (**C‐2**, *λ*_em._ = 601 nm)—are observed for the clusters at 2.4 K indicating the phosphorescent nature of the emission (Figure S27). Consequently, the visible photoluminescence observed for the clusters is attributed to the Bi^3+^‐based ^3^P_1_ → ^1^S_o_ transitions [44, 45]. Other effects reported to induce emission in Bi‐based compounds such as charge transfer (CT), metal‐to‐metal CT, and luminescence from different oxidation states (Bi^2+^, Bi^+^) were reported but are ruled out for the BiO‐NCs [44, 46, 47, 48].

### 2.4 Temperature‐Dependent Photophysical Studies of Eu^3+^ Doped BiO‐NCs

In addition to these photophysical characteristics of the undoped BiO‐NCs the Eu^3+^ doped BiO‐NCs show distinct temperature dependent optical properties, which are similar for all three clusters. Thus **C‐1_D_:Eu** is exemplarily focused on. At 2.4 K, a remarkable excitation wavelength‐dependent emission has been observed for **C‐1_D_:Eu** (Figure 5). Excitation at 314 nm resulted in a dual emission composed of the as‐described Bi^3+^‐based transition and characteristic Eu^3+^‐based ^5^D_0_ → ^7^F*_J_* (*J* = 0 – 4) transitions. Emission monitoring of the broad ^3^P_1_ → ^1^S_0_ band at 556 nm yielded an excitation band with a maximum centered at 314 nm and a shoulder at 336 nm (Figure 5). The maximum is comparable with the ones observed for the undoped clusters **C‐1_D_** and **C‐2** (Figure 4). However, the excitation spectrum obtained by monitoring emission at 611 nm corresponds to the hypersensitive ^5^D_0_ → ^7^F_2_ transition featuring two equally intense bands centered at 314 nm (Bi^3+^‐based transition) and 327 nm (Figure 5). The absence of the band at 327 nm in the undoped cluster and its presence in the Eu^3+^ doped cluster indicates to originate from a O^2–^‐Eu^3+^ ligand‐to‐metal charge‐transfer (LMCT). In addition, Eu^3+^‐based 4f transitions are observed at 360 nm (^7^F_0_ → ^5^D_4_), 376 nm (^7^F_0_ → ^5^G_4_), 393 nm (^7^F_0_ → ^5^L_6_), 464 nm (^7^F_0_ → ^5^D_2_), 525 nm (^7^F_0_ → ^5^D_1_) (Figure 5b and Table S20) [10], in line with the solid‐state UV–vis (DRS) studies (Figure 3 and Table S18).

**FIGURE 5:**
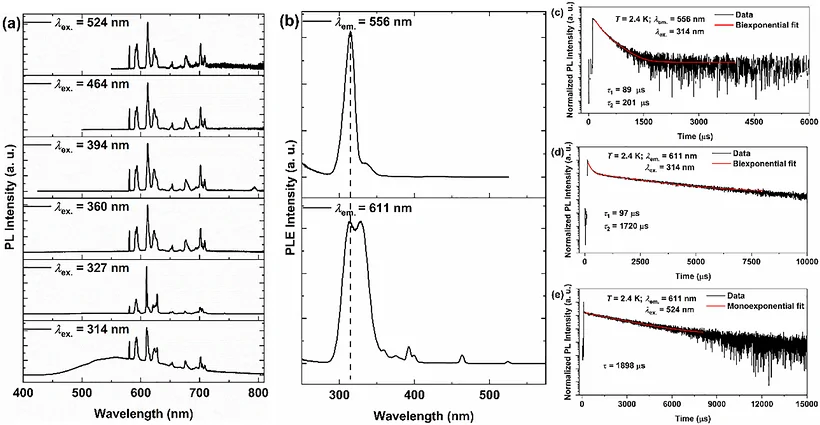
(a) Excitation wavelength‐dependent PL emission spectra of **C‐1_D_:Eu** at 2.4 K (b) PLE spectra of **C‐1_D_:Eu** at 2.4 K. The black dotted line indicates the Bi^3+^‐based ^1^S_0_ → ^3^P_1_ transition. Photoluminescence lifetime decay plot of **C‐1_D_:Eu** at 2.4 K. (c) The emission was monitored at 556 nm under excitation with 314 nm. The decay is fitted with a biexponential function to extract the lifetime (for details refer to Figure S30a). (d) The emission was monitored at 611 nm under excitation with 314 nm. The decay is fitted with a biexponential function to extract the lifetime (for details refer to Figure S30b). (e) The emission was monitored at 611 nm under excitation with 524 nm. The decay is fitted with a mono‐exponential function to extract the lifetime (for details refer to Figure S30c).

In the cases of **C‐2_D_:Eu** and **C‐2:Eu** similar excitation spectra, monitored at 560 nm and 611 nm at 2.4 K, were observed and compared to **C‐1_D_:Eu** (Figure S28). All spectra are dominated by the dual excitation of Bi^3+^‐based transition and O^2—^Eu^3+^ ligand‐to‐metal charge‐transfer, which however is slightly shifted, wider and partially overlapping for **C‐2_D_:Eu** and **C‐2:Eu** indicating a minor influence of the ligand shell. Unlike in the case of **C‐1_D_:Eu**, the dual emission is dominant for the **C‐2_D_:Eu** and **C‐2:Eu** clusters when they are excited around 330 nm. This is attributed to the overlap between the Bi^3+^‐based and LMCT transitions in **C‐2:Eu** and **C‐2_d_:Eu**.

To probe the excitation wavelength dependence of the emission decay at 2.4 K, time‐dependent studies of **C‐1_D_:Eu** were performed by excitation at 314 nm and 524 nm (Figure S30). The decay profile obtained by exciting the cluster at 314 nm and monitoring emission at 556 nm (Figure 5c) can be satisfactorily fitted with a bi‐exponential function and gave lifetimes, which followed the trend observed for the corresponding undoped cluster (**C1_D_**: *τ*_1_ = 65 µs and *τ*_2_ = 143 µs vs. **C1_D_‐Eu**: *τ*_1_ = 89 µs and *τ*_2_ = 201 µs; Figures S27 and 5d). The decay profile obtained by exciting the sample at 314 nm and monitoring emission at 611 nm (Figure 5d) can also be satisfactorily fitted with a bi‐exponential function giving lifetimes of about *τ*_1_ = 96 µs and *τ*_2_ = 1720 µs. The short and the long components are respectively attributed to the Bi^3+^‐ and Eu^3+^‐based transitions. The observation of a Bi^3+^‐based component even while monitoring the emission at 611 nm indicates that the Bi^3+^‐based excited states are not quantitatively quenched via energy transfer to Eu^3+^‐based levels and a significant overlap between the transitions is observed in the emission profile (Figure 5a). Remarkably, the decay profile obtained by exciting the cluster at 524 nm (Figure 5e) can be satisfactorily fitted with a mono‐exponential function yielding a lifetime *τ* = 1898 µs. Such mono‐exponential decay indicates that the Eu^3+^ sites are exclusively excited via inter‐configurational ^7^F_0_ → ^5^D_1_ transition. Similar trends have been observed in the time‐dependent studies of the clusters **C‐2_D_:Eu** and **C‐2:Eu** (Figures S31 and S32) performed at 2.4 K.

To get insights into the emission characteristics, we have systematically studied the emission of **C‐1_D_:Eu** at 2.4 K by excitation at different wavelengths. Unlike in the case of 314 nm excitation, excitation of **C‐1_D_:Eu** at 327 nm resulted in an emission profile dominated by Eu^3+^‐based ^5^D_0_ → ^7^F*_J_* (*J* = 0 – 4) transitions (Figure 5 and Table S21). These facts indicate that firstly the excitation at Bi^3+^‐based transition at 314 nm results into the unquenched Bi^3+^‐based emission and Bi^3+^‐sensitized Eu^3+^‐based emission, secondly excitation of the CT transition at 327 nm promotes mostly sensitized Eu^3+^‐based emission. Excitation at higher wavelengths—360 nm (^7^F_0_ → ^5^D_4_), 376 nm (^7^F_0_ → ^5^G_4_), 393 nm (^7^F_0_ → ^5^L_6_), 464 nm (^7^F_0_ → ^5^D_2_), 524 nm (^7^F_0_ → ^5^D_1_)—gave dominant Eu^3+^‐based ^5^D_0_ → ^7^F_J_ emission. The above observation indicates that Eu^3+^‐based emission can be observed for the cluster upon direct excitation of Eu^3+^‐based inter‐configurational f‐f transitions but also via Bi^3+^ sensitization. Similar emission characteristics have been observed for **C‐2_D_:Eu** and **C‐2:Eu** (Figure S29).

We further had a deeper look on the temperature dependence of photoluminescence profiles of the Eu^3+^ doped BiO‐NCs (Figure S33). On temperature increase to 77 K, PLE, PL, and lifetime profiles for the NCs (Figures S34 and S38) similar to those at 2.4 K have been observed for all Eu^3+^ doped BiO‐NCs. On the other hand, at 180 and 300 K no discernible Bi^3+^‐based transitions are present any more upon exciting the sample at the broad Bi^3+^‐based transitions in the UV region, and only Eu^3+^ based transitions show up (Figures S39–S48). Similarly, for the undoped BiO‐NCs the Bi^3+^‐based phosphorescent transitions are quenched at higher temperatures (>180 K). The lifetime studies of the clusters performed at 77 K followed the trend observed at 2.4 K, and agree with the PLE, PL profiles with regard to the Bi^3+^ and Eu^3+^‐based excitation and emission (Tables S22–S24). Lifetime profiles recorded at 180 and 300 K can be fitted with mono‐exponential decays—irrespective of excitation wavelength—on monitoring emission at 611 nm. In summary, we thus conclude energy transfer (qualitatively) from the excited Bi^3+^ to the Eu^3+^ centres in the doped BiO‐NCs in addition to non‐radiative relaxation on the basis that (i) excitation into the Bi^3^^+^‐centered absorption region leads to Eu^3^^+^ emission, (ii) the excitation profiles monitored at Bi^3^^+^‐ and Eu^3^^+^‐based emission wavelengths are closely comparable, and (iii) Eu^3^^+^ emission is also observed upon direct excitation of Eu^3^^+^‐based transitions in the visible region. This is in line with the absence of Bi^3+^‐based emission at 180 and 300 K for the undoped clusters **C‐1_D_** and **C‐2**. The emission colors of the different Eu^3+^ doped BiO‐NCs, determined from the CIE 1931 diagrams (Figure S49), show similar temperature‐dependent color variation from yellow in agreement with dual emission at 2.4 K and red with absence of Bi^3+^ emission at 300 K.

At 180 and 300 K, the PLE spectra of the Eu^3+^ doped clusters obtained by monitoring emission at 611 nm revealed several interesting facts. The strong bands in the 300 nm to 350 nm region lost intensity relative to the ones observed at cryogenic temperatures. Additionally, we noted a bathochromic shifting of the PLE maxima with the temperature increase (Figures 6 and S50). The intensity of the inter‐configurational f‐f transitions seemingly increase with increasing temperature. However, such increase is likely due to the intensity loss of the Bi^3+^‐based transition. At 180 and 300 K, population of the ^7^F_1_ states is expected considering the small energy gap (about 359 cm^−1^, **C‐1_D_:Eu**; 363 cm^−1^, **C‐2_D_:Eu**; 399 cm^−1^, **C‐2:Eu**) between the ^7^F_0_ and ^7^F_1_ levels. Accordingly, we have observed the emergence of the ^7^F_1_ → ^5^D_1_ (533 nm) and ^7^F_1_ → ^5^D_3_ (416 nm) transitions starting from 180 K (Figures 6 and S50). The estimated energy difference between the ^7^F_0_ → ^5^D_1_ (524 nm; 19084 cm^−1^) and ^7^F_1_ → ^5^D_1_ (533 nm; 18761 cm^−1^) transitions is about 323 cm^−1^, confirming the assignments of F_1_‐based transitions in PLE at 180 and 300 K. The presence of excitation features corresponding to the ^7^F_1_ state at higher temperatures serves as optical evidence for the thermal population of the state. Such observation corroborates well with magnetic studies reporting non‐zero magnetic moments for Eu^3+^ complexes at 300 K [49]. Remarkably, the ^5^D_0_ → ^7^F*_J_* (*J* = 0 – 4) emission can be observed at 300 K (Figure S45) upon direct f‐f excitation via the ^7^F_1_ → ^5^D_1_ and ^7^F_1_ → ^5^D_3_ transitions indicating a photophysical pathway involving fast non‐radiative decay of the ^5^D_1_ state to the ^5^D_0_ state and the subsequent ^5^D_0_ → ^7^F_J_ emission.

**FIGURE 6:**
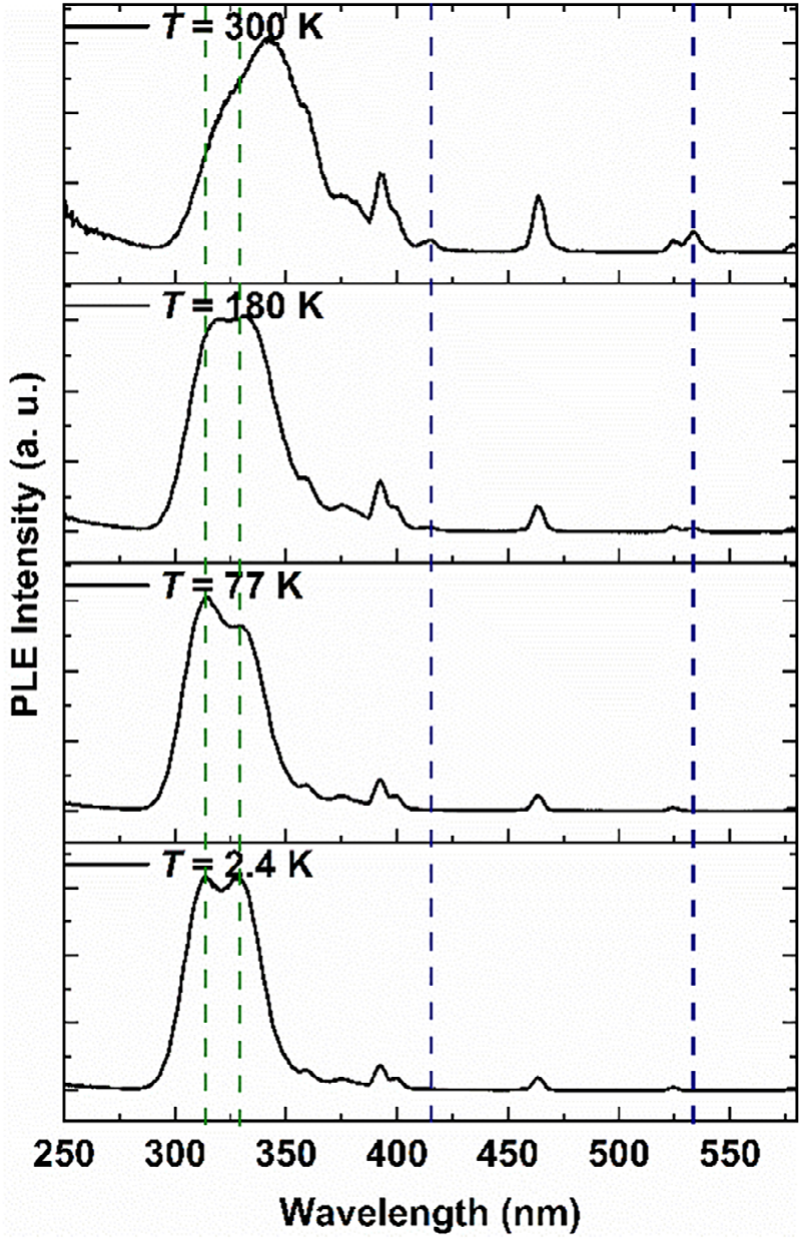
Temperature‐dependent PLE profiles of C‐1_D_:Eu. The green dashed lines indicate the progressive bathochromic shifting of excitation peaks. The blue dashed lines indicate the emergence of the ^7^F_1_ → ^5^D_3_ (416 nm) and ^7^F_1_ → ^5^D_1_ (533 nm) transitions from 180 K.

In the following section we take a closer look on the emission profiles analysis and estimate various parameters to get quantitative insights in the emission process of the Eu^3+^ doped BiO‐NCs. Furthermore, the emission profile can be used to obtain information about the environment of the Eu^3+^ within the BiO‐NCs. For the latter most commonly, the asymmetric integral ratio (R) of the electric dipole (ED)‐induced ^5^D_0_ → ^7^F_2_ transition, highly sensitive to the local coordination, and the magnetic dipole (MD)‐induced ^5^D_0_ → ^7^F_1_ transition, barely influenced by the coordination environment, is determined. In addition, the branching ratio (*β_R_*), and Judd‐Ofelt parameters — Ω2, Ω4, and Ω6 — were determined (Table S25, for details refer to the Supporting Information).

The most intense transition in the clusters is the hypersensitive ^5^D_0_ → ^7^F_2_ transition located at 611 nm, as inferred from the emission profiles and calculated branching ratios (*β_R_*). The second most intense transition is the ^5^D_0_ → ^7^F_4_ transition and the strong intensity of it is attributed to asymmetric coordination environment around Eu^3+^ centers. The calculated asymmetric ratios (R) in the range of 2.4 to 3.4 confirm the nominally distorted coordination environment around Eu^3+^ in the clusters (see coordination polyhedral given in Figures S15 and S22), corroborating well with the presence of a strong ^5^D_0_ → ^7^F_4_ transition. Remarkably, the Judd–Ofelt parameter *Ω4* obtained for the BiO‐NCs is larger than *Ω2* (Table S25) indicating the operation of long‐range effects, such as collective phonon modes or a strong rigidity of the BiO‐NC system. At this point it's worth discussing the strong intensity of the ^5^D_0_ → ^7^F_0_ transition (*λ*_max_ = 580 nm). The 0–0 transition is forbidden by all the selection rules; however, the transition is expected to gain intensity as a consequence of J‐mixing, for example. One of the other factors that might cause J‐mixing is the asymmetric coordination environment around Eu^3+^ in addition to spin‐orbit coupling [10]. However, the strong ^5^D_0_ → ^7^F_0_ and ^5^D_0_ → ^7^F_4_ transitions for the BiO‐NCs might also result from additional mechanisms such as Eu─O and Bi─O charge transfer states mixing with the Eu‐based ^7^F_0_ state and vibronic coupling involving the cluster‐based vibrations in inducing the 0–0 transition. These processes cannot be ruled out at this point. However, the presence of one ^5^D_0_ → ^7^F_0_ transition only and mono‐exponential lifetime decays observed on excitation at 524 nm indicate one emissive Eu^3+^ center or nearly‐equivalent Eu^3+^ centers with minor coordination environment difference. The long lifetimes in the range of 1.8 ms (*λ*_max_ = 611 nm) observed elucidate the presence of the emissive centers in the inner core of the cluster, as their presence at the outer core close to the coordinating ligands such as ^–^OMc and DMSO are expected to shorten the lifetime due to the presence of C─H or O─H oscillators. The long lifetimes observed at cryogenic temperatures and 300 K additionally support the presence of the dopants in the inner part of the cluster core. The barely changing emission spectra with varying ligand shell and coordinating solvent in addition indicate similar ligand field environments around Eu^3+^ in the BiO‐NCs. These results strenghten our hypothesis based on SC XRD of **C1_D_:Eu** and **C2_E_:Eu**, that europium occupies positions mainly in the central {Bi_6_O_9_}:Eu units.

By taking advantage of the measured ^5^D_0_ lifetime, we have quantified the magnitude of non‐radiative decay rate (*k*_nr_) contributing to the Eu^3+^‐luminescence quenching, the radiative lifetime (*τ*_rad_) of the ^5^D_0_ state, the rates of radiative (*k_r_*) and non‐radiative (*k_nr_*) relaxation processes and the intrinsic quantum yields (φ_Eu_) of the clusters (Table 1 details Supporting Information).

**TABLE 1:** Parameters obtained from the PL studies of europium doped BiO‐NCs at 2.4 and 300 K. The BiO‐NCs were excited at 524 nm to avoid the mixing between the Bi^3+^‐ and Eu^3+^‐based emissions.

Parameter	2.4 K			300 K		
	C‐1_D_:Eu	C‐2_D_:Eu	C‐2:Eu	C‐1_D_:Eu	C‐2_D_:Eu	C‐2:Eu
*τ*_rad_	2.94 ms	2.72 ms	3.00 ms	2.95 ms	2.62 ms	2.83 ms
*τ*_obs_	1.98 ms	1.46 ms	1.78 ms	1.64 ms	1.70 ms	1.75 ms
φ_Eu_	65%	54%	59%	57%	65%	62%
*k*_r_	340 s^−1^	368 s^−1^	333 s^−1^	339 s^−1^	382 s^−1^	353 s^−1^
*k_n_*_r_	187 s^−1^	317 s^−1^	234 s^−1^	257 s^−1^	206 s^−1^	217 s^−1^

We observed that the magnitude of *k_r_* is larger than the *k_nr_* at 2.4 and 300 K for the BiO‐NCs (Table 1). This indicates that the emissive ^5^D_0_ state is well‐shielded from the quenching pathways such as O─H and C─H vibrations. Intrinsic quantum yields (φ_Eu_) in the range of 53% to 65% have been obtained for the clusters with a small temperature dependence. The substantial values of φ_Eu_ indicate considerably efficient radiative decay of the ^5^D_0_ state. Please note that the PL signal is stable at least for 20 min under illumination at 314 nm at 300K (Figure S51).

In summary, the combination of the invariant PL signal and the stability of the europium doped BiO‐NCs makes them suitable for use in photoluminescent materials. In order to further evaluate the stability of the europium doped BiO‐NCs, its PL emission properties in solution were investigated representatively using **C‐2_E_:Eu** in ethanol (for details refer to Figure S52). The PL spectra exhibit characteristic Eu^3^^+^ emission bands, albeit with relatively low intensity due to a low europium concentration in solution (c(Eu^3+^) ≈ 10^−^^7^ M).

### 2.5 Photoluminescent Hybrid Materials

The europium doped BiO‐NCs as reported here offer to combine radiopacity, shown previously for undoped BiO‐NCs [50], with unique luminescent properties to give radiopaque photoluminescent organic–inorganic hybrid materials. Especially suitable due to its solubility in common organic solvents and strong PL emission is the europium doped BiO‐NC **C‐2_E_:Eu**. For the synthesis of the hybrid materials, **C‐2_E_:Eu** (*n* = 0.0125 mmol, 0.68 mmol‐%, 2.7·10^−5^ mol%Eu) was used as crosslinking agent in a radical copolymerization of methyl methacrylate (MMA) and 2‐hydroxyethylmethacrylate (HEMA) using AIBN as initiator. Two polymerization approaches were performed, a thermal bulk polymerization leading to a monolithic material and a thermal polymerization of a film as obtained by drop coating of the reaction mixture onto a glass substrate. Both approaches lead to the formation of a transparent polymer (**HM‐C‐2_E_:Eu**) showing an intense red photoluminescence under irradiation with UV light (Figure 7). Noteworthy, approaches for the incorporation of europium complexes and hexanuclear heterobimetallic europium‐titanium oxido clusters in polymeric films as fluorescence agents are described [51, 52, 53, 54]. Here we report on the unique example of incorporation of radiopaque and Eu^3+^ doped NCs with 2 nm in size and stable luminescence.

**FIGURE 7:**
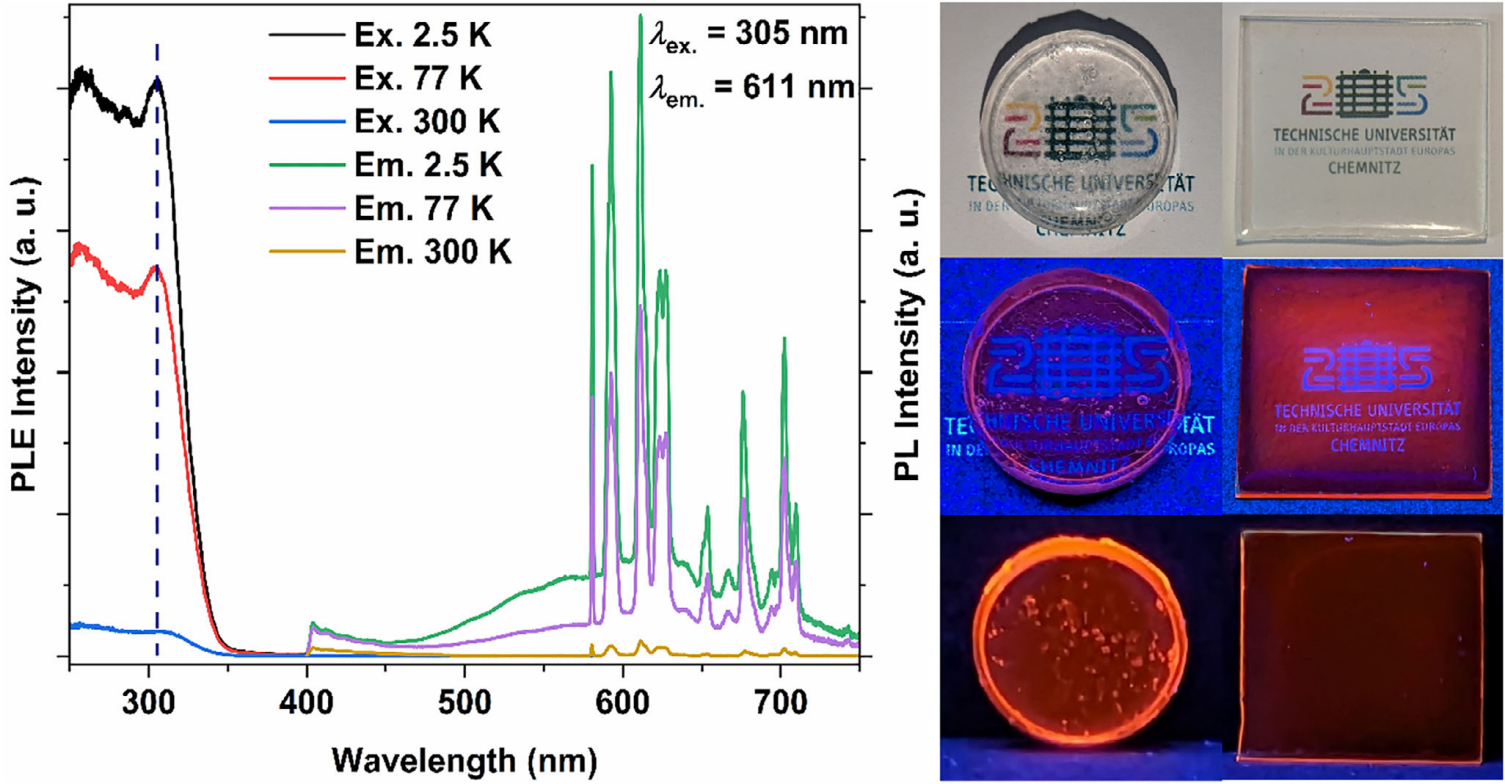
Temperature dependence of PLE and PL of **HM‐C‐2_E_:Eu**. Pictures of the polymer **HM‐C‐2_E_:Eu** monolith (left) and the polymer film at float glass (right). Under sun light with a colored logo (top), under irradiation with UV‐Lamp (*λ* = 254 nm) and with white logo (middle) and under irradiation with UV‐Lamp (*λ* = 254 nm) without logo (bottom).

PL spectroscopy of **HM‐C‐2_E_:Eu** revealed a similar excitation and temperature dependent behavior as found for the nanocluster **C‐2:Eu** itself (for details refer to Figures S53–S61). Excitation spectra showed a broad excitation centered at 305–310 nm, which is slightly shifted compared to the bands of **C‐2:Eu**. Separate peaks for the lanthanide specific excitations were not observed, which is in accordance with respective UV–vis spectra, and is referred to the very low europium content (Figure S62). However, the emission spectra of the hybrid material exhibit a similar emission profile with ^5^D_0_ → ^7^F*_J_* (*J* = 0–4) transitions and temperature dependence as observed for **C‐2:Eu** (Figure S59). At low temperatures dual emission of both Bi^3+^ und Eu^3+^ based PL was observed, but with increased temperature the Bi^3+^ based emission is reduced until its absence at 300 K. The PL emission parameters of **HM‐C‐2_E_:Eu** (for details refer to Tables S26 and S27), such as CIE coordinates, lifetime (*τ* = 2338 µs), asymmetric ratio (*R* = 2.6) and quantum yields (*φ*_Eu_ = 68%) are comparable with the values obtained for **C‐2:Eu**. The PL signal shows only minor decrease of intensity within 20 min under illumination at 314 nm at 300K (Figure S61). Based on similar PL properties of the BiO‐NC **C‐2_E_:Eu** and the hybrid material **HM‐C‐2_E_:Eu** we conclude, the intact BiO‐NC core structure is preserved in the hybrid material. Thus, the Eu^3+^ doped BiO‐NCs are promising candidates for fluorescent materials such as anticounterfeiting markers.

The formation of the polymer materials and the incorporation of the NC **C‐2_E_:Eu** was further proven using ATR–IR spectroscopy (Figure S63). The IR spectra of **HM‐C‐2_E_:Eu** are in a good agreement with the respective neat polymer (**HM**), additionally the Bi–O band at approx. 480 cm^−1^ proves the presence of the BiO‐NC, as reported previously [50]. In addition to promising optical properties as well as radiopacity [50] of the organic–inorganic hybrid material **HM‐C‐2_E_:Eu**, the introduction of the BiO‐NCs lead to improved thermal stability as compared to the pristine polymer, which was demonstrated using thermal analysis under N_2_ and synthetic air (for details refer to Figures S64–S68) of the bulk samples. A delayed decomposition onset by 45 K and complete decomposition delayed by 75 K was observed. We attribute the higher thermal stability of **HM‐C‐2_E_:Eu** (approx. 250°C) to crosslinking by the BiO‐NCs. UV irradiation irradiation (*λ* = 365 nm, LED) for 6 h did not lead to any changes of the IR spectra (Figure S69). In summary, the combination of BiO‐NCs as radiopaque material and the europium doping with a low amount of 2.7·10^−5^ mol%Eu as fluorescence agent lead to transparent red luminescent radiopaque hybrid materials as thin films and monoliths with high thermal stability.

## 3 Conclusion

We have demonstrated the incorporation of Eu^3+^ (≈1 ω%) by doping into the matrix of an atomically precise bismuth oxido nanocluster giving [Bi_38_O_45_(NO_3_)_24_(dmso)_28_]:Eu (**C‐1:_D_Eu**). Ligand exchange reactions starting from **C‐1_D_:Eu** lead to the methacrylate substituted nanoclusters [Bi_38_O_45_(OMc)_24_(dmso)_4_]:Eu·4DMSO·4H_2_O (**C‐2_D_:Eu**), and [Bi_38_O_45_(OMc)_24_(EtOH)_10_]:Eu·4EtOH (**C‐2_E_:Eu**) with about 2 nm in size, showing solubility in organic solvents. Based on a combination of SC‐XRD and photophysical characterization we suggest the main europium doping positions to be placed in the inner {Bi_6_O_9_} core of the clusters, which makes the optical properties less sensitive to environmental effects. Further we demonstrate, that Eu^3+^ doped BiO‐NCs can be used as crosslinkers in transparent organic‐inorganic hybrid materials providing photoluminescent and radiopaque properties and increased thermal stability for **HM‐C‐2_E_:Eu** as compared to the neat polymer material. Most interestingly, all Eu doped BiO‐NCs including the hybrid material show a strong ^5^D_0_ → ^7^F_0_ transition, high asymmetric ratio (for example, *R* = 3.04 for **C‐1:Eu**), lifetimes close to 1.8 ms at 2.4 K and intrinsic quantum yields between 50% to 68%. These alluring properties render the atomically precise europium doped BiO‐NCs suitable fluorescent materials, for example, as used in bioimaging (markers), temperatures sensors, light emitters, LEDs/displays or anticounterfeiting materials. In addition, the strong intensity of the ^5^D_0_ → ^7^F_0_ transition observed as result of the asymmetric coordination sphere of Eu^3+^ render the Eu^3+^ doped clusters as potential materials for implementing photonic quantum information processing schemes as an alternative for recently reported Eu^3+^‐based molecular complexes.

## Author Contributions

**Rico Thomas**: conceptualization, formal analysis, investigation, visualization, Writing – original draft, review and editing. **Senthil Kumar Kuppusamy**: PL investigation, formal analysis, writing – original draft (photophysical part), review and editing, visualization. **Tobias Rüffer**: single‐crystal investigation, formal analysis, writing – review and editing. **Marcus Weber**: ESI–MS investigation, formal analysis, writing – review and editing. **Mario Ruben**: supervision, funding acquisition, resources, writing – review and editing. **Michael Mehring**: supervision, conceptualization, resources, writing – review and editing, funding acquisition. All authors have given approval to the final version of the manuscript.

## Conflicts of Interest

The authors declare no conflicts of interest.

## Supporting information




**Supporting File 1**: Chem71580‐sup‐0001‐SI20260713.docx

## Data Availability

The data supporting our findings in this article has been included as part of the supporting information. In addition CCDC 2523077 (**C‐1_D_:Eu**), CCDC 2523078 (**C‐2_D_:Eu**, sample 1), CCDC 2523079 (**C‐2_D_:Eu**, sample 2), CCDC 2523080 (**C‐2_D_’**), CCDC 2523081 (**C‐2_E_’**), CCDC 2523082 (**C‐2_E_:Eu**, sample 1) and CCDC 2523083 (**C‐2_E_:Eu**, sample 2) contain the supplementary crystallographic data for this paper. These data can be obtained free of charge via www.ccdc.cam.ac.uk/data_request/cif, or by emailing data_request@ccdc.cam.ac.uk, or by contacting The Cambridge Crystallographic Data Centre, 12 Union Road, Cambridge CB2 1EZ, UK; fax: +44 1223 336033. A preprint of this article was previously deposited on *ChemRxiv* [55].
