# Supplementary material for: Europium Doped Atomically Precise Bismuth Oxido Nanoclusters as Molecular Building Blocks for Photoluminescent Hybrid Materials
Source: Chemistry. 2026 Aug 17;32(41):e71580. doi: 10.1002/chem.71580 (PMC13729955; doi:10.1002/chem.71580)
Supplement: Supplementary file 1 [file CHEM-32-e71580-s001.docx]

**Supporting Information**

**Europium Doped Atomically Precise** **Bismuth Oxido Nanoclusters as Molecular Building Blocks for Photoluminescent Hybrid Materials**

*Rico Thomas^a^, Senthil Kumar Kuppusamy^b^, Tobias Rüffer^c^, Marcus Weber^a,d^, Mario Ruben^b,e,f*^ Michael Mehring^a,d*^*

*^a^ Faculty of Natural Sciences, Institute of Chemistry, Coordination Chemistry, Chemnitz University of Technology, Straße der Nationen 62, 09107 Chemnitz, Germany*

*^b^* *Karlsruhe Institute of Technology (KIT), Institute for Quantum Materials and technologies (IQMT), Karlsruhe, 76131,Germany*

*^c^ Faculty of Natural Sciences, Institute of Chemistry, Inorganic Chemistry, Chemnitz University of Technology, Straße der Nationen 62, 09107 Chemnitz, Germany*

*^d^ Center of Materials, Architectures and Integration of Nanomembranes, Chemnitz University of Technology, Rosenbergstraße 6, 09126 Chemnitz, Germany*

*^e^* *Karlsruhe Institute of Technology (KIT), Institute of Nanotechnology (INT), Karlsruhe, 76131, Germany*

*^f^* Centre Européen de Sciences Quantiques (CESQ), Institut de Science et d’Ingénierie Supramoléculaires (ISIS), 67083 Strasbourg, France

***Correspondence: michael.mehring@chemie.tu-chemnitz.de, mario.ruben@kit.edu

**Table of content**

[1. Materials and Methods	3](#_Toc233968471)

[Chemicals:	3](#_Toc233968472)

[Synthesis of [Bi_38_O_45_(NO_3_)_24_(dmso)_28_]:Eu (**C-1_D_:Eu**)	3](#_Toc233968473)

[Synthesis of [Bi_38_O_45_(OMc)_24_(dmso)_4_]:Eu·4dmso·4H_2_O (**C-2_D_:Eu**)	3](#_Toc233968474)

[Synthesis of [Bi_38_O_45_(OMc)_24_(dmso)_4_]:Eu·4dmso·4H_2_O (**C-2_D_:Eu**-**2**)	3](#_Toc233968475)

[Synthesis of [Bi_38_O_45_(OMc)_24_(EtOH)_10_]:Eu·4EtOH (**C-2_E_:Eu)**	4](#_Toc233968476)

[Preparation of Polymer Hybrid Materials:	4](#_Toc233968477)

[Material Characterization	5](#_Toc233968478)

[2. Cluster Characterization	7](#_Toc233968479)

[NMR	7](#_Toc233968480)

[Quantitative Analysis	8](#_Toc233968481)

[ESI-MS	9](#_Toc233968482)

[SC XRD	13](#_Toc233968483)

[General remarks:	13](#_Toc233968484)

[Structure of **C-1:Eu**:	15](#_Toc233968485)

[Structures of **C-2_D_:Eu** and **C-2_D_’**:	18](#_Toc233968486)

[Structure of **C-2_E_:Eu**:	22](#_Toc233968487)

[PXRD	30](#_Toc233968488)

[UV–vis	31](#_Toc233968489)

[Photophysical studies of undoped and Eu^3+^ doped BiO-NCs	31](#_Toc233968490)

[Photophysical studies of undoped-clusters **C-1_D_** and **C-2**	31](#_Toc233968491)

[Lifetime studies of **C-1_D_** and **C-2** at 2.4 K	32](#_Toc233968492)

[Temperature-dependent photophysical studies of the Eu^3+^ doped BiO-NCs	32](#_Toc233968493)

[Photoluminescence excitation (PLE), photoluminescence (PL), and lifetime studies of the Eu^3+^ doped BiO-NCs at 2.4 K	32](#_Toc233968494)

[Lifetime studies of the Eu doped clusters at 2.4 K	34](#_Toc233968495)

[Compilation of the PLE and PL profiles of the Eu^3+^ doped BiO-NCs ranging from 2.4 K to 300 K	37](#_Toc233968496)

[Photoluminescence excitation (PLE), photoluminescence (PL), and lifetime studies of the Eu^3+^ doped BiO-NCs at 77 K	38](#_Toc233968497)

[Photoluminescence excitation (PLE), photoluminescence (PL), and lifetime studies of the Eu^3+^ doped BiO-NCs at 180 K	41](#_Toc233968498)

[Photoluminescence excitation (PLE), photoluminescence (PL), and lifetime studies of the Eu^3+^ doped BiO-NCs at 300 K	44](#_Toc233968499)

[CIE coordinates of Eu^3+^ doped BiO-NCs at 2.4 K and 300 K	46](#_Toc233968500)

[Compilation of the PLE profiles of the Eu^3+^ doped BiO-NCs ranging from 2.4 K to 300 K	48](#_Toc233968501)

[3. Hybrid Material Characterization	51](#_Toc233968502)

[Temperature-dependent steady-state and time-dependent photoluminescence studies of **HM-C-2_E_:Eu**	51](#_Toc233968503)

[PL, PLE, and lifetime studies of **HM-C-2_E_:Eu** at 77 K	52](#_Toc233968504)

[PL, PLE, and lifetime studies of **HM-C-2_E_:Eu** at 300 K	53](#_Toc233968505)

[Temperature-dependent PL and PLE of **HM-C-2_E_:Eu** at 2.4 K	54](#_Toc233968506)

[UV–vis Hybrid Material	56](#_Toc233968507)

[IR Hybrid Materials	56](#_Toc233968508)

[TGA	56](#_Toc233968509)

[DSC	60](#_Toc233968510)

IR after Irradiation………………………………………………………………………………... 61

[References:	62](#_Toc233968511)

# 1. Materials and Methods

Chemicals: Bi(NO_3_)_3_**·**5H_2_O (98 %) was purchased from Alfa Aesar and Eu(NO_3_)_3_**·**6H_2_O (99.5 %) from Chempur. Sodium methacrylate (NaOMc, 99 %) was purchased from Sigma Aldrich, sodium hydroxide (99 %) from Merck and 2,2’-Azobis(2-methylpropionitril) (AIBN, 99.8 %) from Acros Organics. All chemicals were used without further purification. The solvent dmso (99.9 %, Fisher chemicals) and ethanol (99.9 %, Alfa Aesar) were used without further purification. Iso-propanol (99.95 %, MS Grade) from Carl Roth and dmso (99.5 %, MS Grade) from Thermo Scientific were used without further purification for the ESI-MS measurements. 2-hydroxyethyl methacrylate (HEMA, 97 % Alfa Aesar), methyl methacrylate (MMA, 99 %, Carl Roth) were freshly distilled prior to the polymerization experiments.

[Bi_38_O_45_(NO_3_)_20_(dmso)_28_](NO_3_)_4_**·**4dmso (**C-1_D_**),^1^ [Bi_38_O_45_(OMc)_24_(dmso)_9_]·2dmso·7H_2_O (**C-2_D_**),^2^ and [Bi_38_O_45_(OMc)_24_(EtOH)_13_] (**C-2_E_**)^3^ were synthesised according to literature procedures.

## Synthesis of [Bi_38_O_45_(NO_3_)_24_(dmso)_28_]:Eu (**C-1_D_:Eu**)

Bi(NO_3_)_3_**·**5H_2_O (1504 mg; 3.100 mmol) and Eu(NO_3_)_3_**·**6H_2_O (133 mg; 0.310 mmol) were dissolved with vigorous stirring in dmso (70 mL) under ambient conditions. Subsequently an aqueous NaOH solution (0.31 M, 12 mL) was added dropwise to the solution avoiding turbidity. The reaction mixture was heated to 80 °C for 4 h, followed by a filtration of the hot solution. Gas phase diffusion of acetone in the filtered reaction mixture results in the formation of colorless crystals (352 mg, 0.029 mmol, *η =* 35.1 % based on bismuth) after 4 weeks. Leaving the crystals under ambient conditions results in partial loss of dmso and the formation of a microcrystalline powder.

Elemental analysis CHNS %, exp. and (calcd.) for Bi_37.17_Eu_0.83_O_145_N_24_C_56_H_168_S_28_ (M = 12289.74 g·mol^−1^): C, 5.63 (5.47); H, 1.42 (1.38); N, 2.60 (2.73); S, 7.52 (7.31). ICP-OES (ω%, exp./calcd.): Eu, 0.87 - 1.17 (1.02); Bi/Eu = 1: 0.018-0.029. ATR-IR (cm^–1^): 3700-3100 w, 3002 w, 2915 w, 1740 w, 1642 w, 1431 m, 1381 s, 1267 s, 1000 s, 923 m, 816 m, 706 m, 489 s.

## Synthesis of [Bi_38_O_45_(OMc)_24_(dmso)_4_]:Eu·4dmso·4H_2_O (**C-2_D_:Eu**)

[Bi_38_O_45_(NO_3_)_24_(dmso)_28_]:Eu (**C-1_D_:Eu**, 2000 mg, 0.200 mmol) was dissolved in 35 mL dmso under stirring at 80 °C for 1 h. NaOMc (*m* = 633 mg, *n* = 5.864 mmol) was added to the colorless solution and the mixture was kept for 4 h at 80 °C. The hot solution was filtered subsequently and after two weeks under ambient conditions, colorless crystals of [Bi_38_O_45_(OMc)_24_(dmso)_4_]:Eu·4dmso·4H_2_O (**C-2_D_:Eu**, 1346 mg, 0.163 mmol, *η =* 81.5 % based on bismuth in **C-1_D_:Eu**) were obtained.

Elemental analysis CHNS %, exp. and (calcd.) for (Bi_37.34_Eu_0.66_O_103_C_108_H_164_S_6_, *M* *=* 11206.98 g·mol^–1^): C 11.46 (11.57); H 1.54 (1.47); S 1.68 (1.72). ICP-OES (ω%, expt/calcd): Eu, 0.83 - 0.94 (0.88); Bi/Eu =1: 0.018-0.020. ^1^H NMR (500.30 MHz, dmso-d_6_, 298 K): *δ =* 5.76 (s, 1 H, CH^A^*H*^B^), 5.26 (s, 1 H, C*H*^A^H^B^), 3.36 (s, H_2_O), 2.54 (s, (H_3_C)_2_SO), 1.78 (s, 3 H, CH_3_).^13^C NMR (125.81 MHz, dmso-d_6_, 300 K): *δ =* 173.5, 141.6, 121.2, 40.4 ((H_3_C)_2_SO), 19.7 ATR-IR (cm^–1^): 3700-3100 w, 3090 w, 2968 w, 2919 w, 1641 m, 1518 s, 1450 m, 1397 m, 1375 s, 1359 s,1229 s, 1012 s, 1001 s, 943 s, 926 s, 845 m, 831 s, 703 w, 661 w, 489 s.

## Synthesis of [Bi_38_O_45_(OMc)_24_(dmso)_4_]:Eu·4dmso·4H_2_O (**C-2_D_:Eu**-**2**)

[Bi_38_O_45_(OMc)_24_(EtOH)_14_]:Eu (**C-2_E_:Eu**, 0.280 g, 0.026 mmol) was dissolved in 2.5 mL dmso under stirring at 80 °C for 1 h. The hot solution was filtered subsequently and after two weeks under ambient conditions, colorless crystals of [Bi_38_O_45_(OMc)_24_(dmso)_4_]:Eu·4dmso·4H_2_O (**C-2_D_:Eu**-**2**) suitable for SCXRD were obtained. ICP-OES **C-2_D_:Eu**-**2** (ω%, expt): Eu, 1.01; Bi/Eu =1: 0.023. Please note that the resulting amount of cluster **C-2_D_:Eu**-**2** was only sufficient for analysis using SC XRD and ICP-OES.

## Synthesis of [Bi_38_O_45_(OMc)_24_(EtOH)_10_]:Eu·4EtOH (**C-2_E_:Eu)**

[Bi_38_O_45_(OMc)_24_(dmso)_4_]:Eu·4dmso·4H_2_O (**C-2_D_:Eu**, 1250 mg, 0.111 mmol) was dissolved in 10 mL ethanol under stirring at 60 °C for 1.5 h. The hot solution was filtered subsequently and first allowed to cool to room temperature, and then cooled to 4 °C. After two days, colorless crystals of [Bi_38_O_45_(OMc)_24_(EtOH)_10_]:Eu·4EtOH (**C-2_E_:Eu**, 530 mg, 0.047 mmol, *η =* 42.4 % based on bismuth in **C-2_D_:Eu**,) were obtained. The crystals of **C-2_E_:Eu** were collected and dried in the vacuum at 60 °C for 1 h to remove the EtOH and to give **C-2:Eu**.

Elemental analysis CHNS %, exp. and (calcd.) for **C-2:Eu** (Bi_37.32_Eu_0.68_O_93_C_96_H_120_, *M* *=* 10664.41 g·mol^–1^): C 10.46 (10.81); H 1.19 (1.14). ICP-OES **C-2:Eu** (ω%, expt/calcd): Eu, 0.80 - 1.14 (0.97); Bi/Eu =1: 0.016-0.022. ^1^H NMR (500.30 MHz, CDCl_3_, 298 K): *δ =* 5.81 (s, 1 H, CH^A^*H*^B^), 5.30 (s, 1 H, C*H*^A^H^B^), 3.77 (s, EtOH, OH), 3.69 (q, 2 H EtOH (CH_2_)), 1.82 (s, 3 H, CH_3_), 1.19 (q, EtOH (CH_3_)).^13^C NMR (125.81 MHz, dmso-d_6_, 300 K): *δ =* 176.0, 140.6, 123.5, 58.3 (EtOH), 19.5, 18.6(EtOH). ATR FTIR (cm^–1^): 3700-3100 w, 3091 w, 2969 w, 2921 w, 1640 m, 1505 s, 1450 m, 1398 m, 1377 s, 1360 s,1228 s, 1001 s, 928 s, 844 m, 831 s, 655 w, 484 s.

## Preparation of Polymer Hybrid Materials:

**HM-C-2_E_:Eu:** [Bi_38_O_45_(OMc)_24_(EtOH)_10_]:Eu·4EtOH (**C-2_E_:Eu,** 134 mg, 0.013 mmol) was dissolved in HEMA (1.03 mL, 8.200 mmol) under stirring at 70 °C. After 10 minutes, a clear solution was formed to which MMA (1.06 mL, 10.000 mmol) was added and the temperature was reduced to 60 °C. After a further 10 min of stirring AIBN (16 mg, 0.100 mmol) was added. The homogenous solution was collected with a syringe and transferred into a small glass vial (**HM-C-2_E_:Eu** - monolith) or drop-coated on a float glass substrate (**HM-C-2_E_:Eu** - film). Afterwards the resulting samples were heated for 2 h at 100 °C to give transparent polymer hybrid materials. For analysis of the hybrid material a powdered sample of the monolith (**HM-C-2_E_:Eu**) was used.

Elemental analysis CHNS %, exp. for **HM-C-2_E_:Eu**: C 52.43; H 7.38; N 0.25. ATR FTIR (cm^–1^): 3650-3050 m, 2987 w, 2949 m, 2881 w, 1715 s, 1639 w 1482 w, 1451 m, 1385 m, 1366 w, 1322 w,1267 m, 1243m, 1150 s, 1072 s,1023 m, 945 m, 898 m, 847 m, 749 m, 514 s.

**HM:** In a flask HEMA (1.03 mL, 8.200 mmol) and MMA (1.06 mL, 10.000 mmol) were mixed under stirring at 60 °C. After 10 min of stirring AIBN (16 mg, 0.100 mmol) was added and stirred for another 10 min. The homogenous solution was collected and transferred into a small glass vial using a syringe. Afterwards the resulting sample was heated for 2 h at 100 °C and another 1 h at 150 °C to give a transparent polymer material.

Elemental analysis CHNS %, exp. for **HM**: C 55.95; H 7.80; N 0.30. ATR FTIR (cm^–1^): 3600-3100 m, 2987 w, 2952 m, 1719 s, 1639 w 1478 w, 1448 m, 1388 m, 1265 m, 1242 m, 1146 s, 1073 s,1023 m, 955 m, 902 m, 844 m, 748 m.

## Material Characterization

Powder X-ray diffractograms were measured at ambient temperature with a STOE *Stadi P* diffractometer (Darmstadt, Germany) using Ge(111)-monochromatized Cu-K_α_ radiation (λ = 1.54056 nm, 40 kV, 40 mA). The full width at half maximum (FWHM) is corrected for instrumental broadening using a LaB_6_ standard (*SRM 660*) purchased from the National Institute of Standards and Technology (NIST). The value of *β* was corrected from *β*^2^ = *β*_measuerd_^2^ – *β*_instrument_^2^, where *β*_measuerd_^2^ and *β*_instrument_^2^ are the FWHMs of measured and standard profiles, respectively. SC X-RAY diffraction was performed using Bruker Venture D8 diffractometer including the APEX software package for **C-2_D_:Eu**, **C-2_D_’** and **C-2_E_:Eu**-**1**. The SC X-RAY diffraction of **C-1_D_:Eu** was measured and analyzed using a Rigaku-Oxford Gemini S diffractometer. ^1^H and ^13^C^1^{H} NMR spectra were recorded at room temperature in CDCl_3_ (dried over 4 Å molecular sieve) with an *Avance III 500* spectrometer (Bruker) at 500.30  MHz and 125.81 MHz, respectively, and are referenced internally to the deuterated solvent relative to Si(CH_3_)_4_ (*δ* = 0.00 ppm). The NMR spectra were processed using the software *MestReNova* /version (11.0.4‑18998). CHNS analyses were performed with a Foss Heraeus *Vario EL* analyzer. Infrared spectra were recorded with a *Nicolet iS 5 FT-IR (Fourier Transformation Infrared Spectroscopy)* spectrometer (Thermo Scientific) with an *iD7 AR-coated diamond crystal ATR* accessory. Solid samples were pressed with 40 pounds onto the crystal. Spectra were recorded with 32 scans and a resolution of 4 cm^−1^ using the Omnic 9 software (version 9.8.372). UV–vis spectroscopy was performed using a *Carry 60 UV–vis* spectrometer (Agilent Technologies) equipped with a *Barrelino*™ (Harrick Scientific Products) remote diffuse reflection probe. The particle size distribution (PSD) was determined using a *Zetasizer Nano ZS* (Malvern Instruments) with dynamic light scattering (DLS). Using laser light (*λ* = 633 nm, *P* = 4 mW) as a light source, suspensions with a size range from 0.4 nm to 6 μm can be investigated. The analyses were performed at an angle of 173 ° (non-invasive backscatter (NIBS) default). The powders were dissolved in different solvents (*β* = 20 g·L^–1^) and filled into glass cuvettes (DTS0012) and measured at *T* = 20 °C. The calculation of the PSD was automatically performed following the “Mie theory” assuming spherical particles. Metal content analysis was conducted via inductively-coupled plasma – optical emission spectroscopy (ICP-OES), with a VistaRL (VARIAN). 10 mg of the samples were dissolved in 5 mL nitric acid (65 %) and diluted to 10 vol‑% acid with ultrapure water. Photophysical studies of the undoped- and Eu^3+^ doped BiO-NCs were performed using a Horiba quantum master spectrometer with a R920 photomultiplier tube detector. The gently ground samples were placed in-between two quartz plates with a drop of perfluorinated oil and mounted on a Sumitomo closed-cycle He-cryostat for temperature-dependent measurements in the range from 2.4 K to 300 K. A 400 nm long pass filter was used to cut out the second- and higher-order diffraction peaks in the spectra. Correction files supplied by the manufacturer were used to obtain the corrected emission spectra. The data plotting and decay fitting were performed using Origin 24. CIE coordinates were calculated using the CIE-coordinate app in Origin 24. Fluorescence measurements of **C-2_E_:Eu** in ethanol solution (*c* = 0.0001 M) were performed with a Horiba Jobin Yvon *FluoroMax* spectrofluorometer. Measurements of the emission spectra were conducted in quartz glass cuvettes and recorded at different excitation wavelengths (*λ*_ex_= 350 nm, *λ*_ex_= 395 nm, *λ*_ex_= 464 nm) with a slit width of 5 nm. Electrospray ionization mass spectrometry (ESI-MS) was carried out using a Bruker Trapped Ion Mobility Spectrometry (tims) time-of-flight (TOF) mass spectrometer (Bruker Daltonik GmbH). Calibration was performed in the *m*/*z* range of 100 – 10000 using cesium perfluoroheptanoate (*c* = 5 mM in H_2_O/MeCN with *V*:*V* = 1:1, abcr GmbH). The crystals of compound **C-2_D_:Eu** and **C-2_E_:Eu** were dissolved in iso-propanol (ultra LC-MS grade, Carl Roth) with a final concentration of 100 µM, respectively. The crystals of **C-1_D_:Eu** were dissolved in dmso (LC-MS grade, purity by titration: 99.7 %, water content: ≤ 0.1 %, thermo scientific) to give a final concentration of 100 μM. The sample was injected into the ESI source using a Hamilton syringe (*V* = 500 µL) at a flow rate of 180 μL·h^−1^. The voltage of the spray capillary was set to 4.5 kV (positive mode) with deflection delta of 70.0 V and an end plate offset voltage of 500 V. The dry gas flow was about 3.0 L·min^−1^ and a drying temperature of 200 °C was used. Mass spectra were treated by baseline subtraction (flatness 0.3) and smoothing (Savitzky Golay algorithm, width 0.05 *m*/*z*). Isotope patterns were calculated using Bruker Compass Data Analysis software (Copyright © 2023 Bruker Daltonik GmbH, version 6.1). DSC experiments were conducted from 30 °C – 500 °C on a DSC 1 calorimeter (Mettler Toledo) using sealed 40 μL aluminum crucibles under N_2_ atmosphere and a heating rate of 10 K·min^−1^. TGA measurements were performed in the range of 30 °C – 900 °C on a *TGA/DSC3+* with a XP1U balance (HTSO801RO / GC401, Mettler Toledo) in uncapped 70 μL aluminum oxide crucibles under N_2_ or air flow and a heating rate of 10 K·min^−1^.

# 2. Cluster Characterization

## NMR

Figure S 1. ^1^H NMR spectra (500.3 MHz, 298 K) of **C-2_D_:Eu** in dmso-d_6_. **(b)** ^13^C NMR spectra (125.8 MHz, 298 K) of **C-2_D_:Eu** in dmso-d_6_.

Figure S 2. ^1^H NMR spectra (500.3 MHz, 298 K) of **C-2_E_:Eu** in CDCl_3_. The additional signal marked with * belongs to residuals of DMSO and the coordinated ethanol signals are marked with the groups (OH, CH_2_, CH_3_). **(b)** ^13^C NMR spectra (125.8 MHz, 298 K) of **C-2_E_:Eu** in CDCl_3_ . Signals 5 and 6 belong to ethanol.

## Quantitative Analysis

Table S 1. Summary of the europium content values and the Eu/Bi ratios of the BiO-NCs **C-1:Eu** – **C-2_E_:Eu** determined with ICP-OES (multiple determination).

| **BiO-NCs** | **ICP-OES Eu [ω%]** | **ICP-OES Eu/Bi** |
| --- | --- | --- |
| **C-1_D_:Eu** | 0.87(3) – 1.17(3) | 0.018 – 0.029 |
| **C-2_D_:Eu** | 0.83(2) – 0.94(2) | 0.018 – 0.020 |
| **C-2:Eu** | 0.80(15) – 1.14(3) | 0.016 – 0.022 |

## ESI-MS

ESI-MS spectra of all doped clusters were analysed, assigned are the most abundant and differently doped fragments to the respective cationic species, since the spectra do not significantly differ from the undoped clusters.^4-5^

Figure S 3. Survey ESI-MS spectra generated from cluster **C-1_D_:Eu** after electrospraying from dmso, with marked doubly, triply and quadruply positive charged species ranges.

Figure S 4. Part of the mass spectrum of compound **C-1_D_:Eu** electrosprayed from dmso showing quadruply positive charged homo- (**I** – **III**) and heterometallic (**IV** - **VIII**) BiO-NC cations with [Bi_38_O_46_(NO_3_)_18_(dmso)_7_]^4+^ (**II**) at *m*/*z* = 2584.7243 showing the highest abundance.

Table S 2. Selection of quadruply positively charged BiO-NC cations detected in the survey mass spectrum of BiO-NC **C-1:Eu** electrosprayed from dmso. Assignment was carried out using the most abundant m/z signals.

| **Label** | **Cation** | ***m*/*z*** | |
| --- | --- | --- | --- |
|  |  | **calculated** | **BiO-NC C-1_D_:Eu** |
| **I** | [Bi_38_O_45_(NO_3_)_20_(dmso)_8_]^4+^ | 2631.2230 | 2631.2207 |
| **II** | [Bi_38_O_46_(NO_3_)_18_(dmso)_7_]^4+^ | 2584.7243 | 2584.7249 |
| **III** | [Bi_38_O_46_(NO_3_)_18_(dmso)_6_]^4+^ | 2565.2208 | 2565.2231 |
| **IV** | [Bi_36_Eu_2_O_46_(NO_3_)_18_(dmso)_7_]^4+^ | 2556.1943 | 2556.1984 |
| **V** | [Bi_37_EuO_46_(NO_3_)_18_(dmso)_6_]^4+^ | 2551.2061 | 2551.2092 |
| **VI** | [Bi_36_Eu_2_O_46_(NO_3_)_18_(dmso)_6_]^4+^ | 2536.6908 | 2536.6950 |
| **VII** | [Bi_35_Eu_3_O_46_(NO_3_)_18_(dmso)_6_]^4+^ | 2522.6760 | 2522.6816 |
| **VIII** | [Bi_34_Eu_4_O_46_(NO_3_)_18_(dmso)_6_]^4+^ | 2508.6611 | 2508.6706 |

Figure S 5. Isotopic patterns (exp. and calcd.) of different quadruply positive BiO-NC species detected in the gas phase after electrospraying of compound **C-1_D_:Eu** from dmso. [Bi_38_O_45_(NO_3_)_20_(dmso)_8_]^4+^ (**I**), [Bi_38_O_46_(NO_3_)_18_(dmso)_7_]^4+^ (**II**), [Bi_38_O_46_(NO_3_)_18_(dmso)_6_]^4+^ (**III**), [Bi_36_Eu_2_O_46_(NO_3_)_18_(dmso)_7_]^4+^(**IV**), [Bi_37_EuO_46_(NO_3_)_18_(dmso)_6_]^4+^ (**V**), [Bi_36_Eu_2_O_46_(NO_3_)_18_(dmso)_6_]^4+^(**VI**), [Bi_35_Eu_3_O_46_(NO_3_)_18_(dmso)_6_]^4+^ (**VII**), [Bi_34_Eu_4_O_46_(NO_3_)_18_(dmso)_6_]^4+^(**VIII**).

Figure S 6. Survey ESI-MS spectrum generated from BiO-NC **C-2_D_:Eu** after electrospraying from *^i^*PrOH, with marked doubly and triply positive charged species ranges.

Figure S 7. Part of the mass spectrum of compound **C-2_D_:Eu** electrosprayed from *^i^*PrOH showing doubly positive charged homo- (**IX** – **X**) and heterobimetallic (**XI** – **XIV**) BiO-NC cations with [Bi_38_O_45_(OMc)_22_]^2+^ (**IX**) at *m*/*z* = 5265.8311 showing the highest abundance.

Figure S 8. Survey ESI-MS spectra generated from cluster **C-2_E_:Eu** after electrospraying from *^i^*PrOH, with marked doubly and triply positive charged species ranges.

Figure S 9 Part of the mass spectrum of compound **C-2_E_:Eu** electrosprayed from *^i^*PrOH showing doubly positive charged homometallic ([Bi_38_O_45_(OMc)_22_]^2+^(**IX**), [Bi_38_O_45_(OMc)_21_(O*i*Pr)]^2+^ (**X**)) and heterobimetallic ([Bi_37_EuO_45_(OMc)_22_]^2+^ (**XI**), [Bi_36_Eu_2_O_45_(OMc)_22_]^2+^ (**XII**), [Bi_35_Eu_3_O_45_(OMc)_22_]^2+^ (**XIII**), [Bi_34_Eu_4_O_45_(OMc)_22_]^2+^ (**XIV**)) BiO-NC cations.

Table S 3. Selection of doubly positively charged BiO-NC cations detected in the survey mass spectrum of BiO-NCs **C-2:Eu** and **C-2_E_:Eu** electrosprayed from *^i^*PrOH, respectively. Assignment was carried out using the most abundant *m*/*z* signals.

| **Label** | **Cation** | ***m*/*z* (calculated)** | | |
| --- | --- | --- | --- | --- |
|  |  | **calculated** | **BiO-NC C-2_E_:Eu** | **BiO-NC C-2_D_:Eu** |
| **IX** | [Bi_38_O_45_(OMc)_22_]^2+^ | 5265.8311 | 5265.8376 | 5265.8284 |
| **X** | [Bi_38_O_45_(OMc)_21_(O*i*Pr)]^2+^ | 5252.8415 | 5252.8432 | 5252.8367 |
| **XI** | [Bi_37_EuO_45_(OMc)_22_]^2+^ | 5237.8015 | 5237.8102 | 5237.7998 |
| **XII** | [Bi_36_Eu_2_O_45_(OMc)_22_]^2+^ | 5209.7719 | 5209.7813 | 5209.7693 |
| **XIII** | [Bi_35_Eu_3_O_45_(OMc)_22_]^2+^ | 5180.7427 | 5180.7503 | 5180.7398 |
| **XIV** | [Bi_34_Eu_4_O_45_(OMc)_22_]^2+^ | 5152.7134 | 5152.7205 | 5152.7109 |

Figure S 10. Isotopic patterns (exp. and calcd.) of different doubly positive charged BiO-NC cations detected in the gas phase generated from compound **C-2_D_:Eu** after electrospraying from *^i^*PrOH. [Bi_38_O_45_(OMc)_22_]^2+^ (**IX**), [Bi_38_O_45_(OMc)_21_(O*i*Pr)]^2+^ (**X**), [Bi_37_EuO_45_(OMc)_22_]^2+^(**XI**), [Bi_36_Eu_2_O_45_(OMc)_22_]^2+^ (**XII**), [Bi_35_Eu_3_O_45_(OMc)_22_]^2+^ (**XIII**), [Bi_34_Eu_4_O_45_(OMc)_22_]^2+^(**XIV**).

Figure S 11. Isotopic patterns (exp. and calcd.) of different doubly positive charged BiO-NC species detected in the gas phase generated from compound **C-2_E_:Eu** after electrospraying from *^i^*PrOH. [Bi_38_O_45_(OMc)_22_]^2+^ (**IX**), [Bi_38_O_45_(OMc)_21_(O*i*Pr)]^2+^ (**X**), [Bi_37_EuO_45_(OMc)_22_]^2+^ (**XI**), [Bi_36_Eu_2_O_45_(OMc)_22_]^2+^ (**XII**), [Bi_35_Eu_3_O_45_(OMc)_22_]^2+^ (**XIII**), [Bi_34_Eu_4_O_45_(OMc)_22_]^2+^(**XIV**).

## SC XRD

### General remarks:

Europium content determination: Significance of the Eu^3+^ doping in the lanthanide doped BiO-NCs was tested in accordance to a previously reported procedure.^6-7^ Initially we calculated for each individual Bi atomic position whether it is occupied by Eu^3+^, or not. We used EXYZ and EADP constrains to fix atoms to one and the same position and used a free parameter for refinement of the europium content. In case that the thus calculated europium content did not exceed significantly its standard deviation with respect to the 3σ criteria,^8^ we assume this position being occupied by europium (at least to a minor extent). This refers to the compounds denoted as **C-1_D_:Eu**, **C-2_D_:Eu-1**, **C-2_D_:Eu-2**, **C-2_E_:Eu-1**, **C-2_E_:Eu-2**. Crystallographic information files (.cif files) of these compounds/refinements have been deposited with the CSD database.

Determination of the significance of europium doping/content: In order to increase the statistical reliability and to eliminate potential interference effects with respect to the Eu^3+^ occupancy we took the final .ins file of the final refined doped BiO-NC and performed another refinement against the .hkl file of the undoped BiO-NC. In selected cases that lead to the exclusion of Eu^3+^ doping at certain Bi positions (3σ criteria, see below in more detail). In any case the thus obtained Eu^3+^ contents were subtracted from the initially refined ones. This procedure provides a significance test and gives modified values of the Bi to Eu contents. **Please notice:** The thus obtained corrected values are discussed within this work, but have not been deposited with the CSD database.

Bond length criteria: In accordance to previously published BiO-NC structures, we observed several M–O distances which are longer than the sum of the covalent radii (2.14 Å (Bi–O) and 2.64 Å (Eu–O)),^9^ but which are significantly below the sum of the *van der Waals* radii of 3.59 Å (Bi^…^O) and 3.87°Å (Eu^…^O).^10-12^ Especially with respect to Alvarez and the crystallographic characterization of many further “Bi^…^O” and “Eu^…^O” (^…^ refers to any type of interaction/bond) containing compounds we aimed to derive a criterion up to which bismuth oxygen and europium oxygen contacts should be considered as interacting.

We thus analyzed the Bi^…^O bond distances of 11703 entries from the CCDC ConQuest (version 2022.1.0) in order to estimate an upper bond distance limit (Figure S 12). The Bi^…^O bonds range between 1.757 Å^13^ and 3.429 Å^14^, and the median of the bond length was determined with 2.418 Å and the upper and lower quartile with 2.622 Å and 2.259 Å, respectively. Similarly, the Eu^…^O bond distances of 4494 entries from the CCDC ConQuest (version 2022.1.0 Database 2021/11) were analyzed in order to estimate an upper bond distance limit (Figure S 12). The Eu^…^O bonds range between 1.913 Å^15^ and 3.114 Å^16^, the median of the bond length was determined with 2.418 Å and the upper and lower quartile with 2.479 Å and 2.368 Å, respectively.

Both M^…^O bond histograms (Figure S 12) show hardly any entries above a length above 3.15 Å, which we took as the upper limit for both Bi–O and Eu–O bonding interactions. The upper limit includes more than 99.8% of all entries. **Please notice**: “M^…^O” referred so far in this discussion to the bond type, by which an atom M is bonded by any type to an O atom, according to CSD search routines. We refer from here on to “M–O”, which includes any bond types without further differentiation. In a few specific cases the application of this limit of 3.15 Å leads to coordination polyhedra with inadequate coordination numbers and unrealistic coordination geometries (Figure S 22). In such cases and as mentioned explicitly below we increased the distance criteria to 3.59 Å Bi^…^O and 3.87 Å Eu^…^O, corresponding to the sum of the *van der Waals* radii.

Figure S 12. Histogram of bond lengths of Eu^…^O (*left*) and Bi^...^O (*right*) based on CCDC ConQuest determination.

### Structure of **C-1:Eu**:

Table S 4. SC XRD crystallographic data for [Bi_38_O_45_(NO_3_)_24_(dmso)_28_]:Eu (**C-1_D_:Eu**), [Bi_38_O_45_(NO_3_)_24_(dmso)_28_](NO_3_)_4_·4dmso (**C-1**)^1^.

|  | **C-1_D_:Eu** | **C-1_D_^1^** |
| --- | --- | --- |
| Empirical formula | Bi_37_._26_Eu_0_._74_O_145_C_56_H_168_N_24_S_28_ | Bi_76_O_294_C_120_H_360_N_48_S_60_ |
| *M* / g·mol^–1^ | 12294.74 | 24986.64 |
| *T* / K | 100 | 100 |
| *λ* / Å | 0.71073 | 0.71073 |
| Space group | *C2/c* | *C2/c* |
| *a* / Å | 29.6095(4) | 29.6563(2) |
| *b* / Å | 27.7697(4) | 27.7650(2) |
| *c* / Å | 29.1825(4) | 29.1273(3) |
| *α* / ° | 90 | 90 |
| *β* / ° | 92.806(1) | 92.667(1) |
| *γ* / ° | 90 | 90 |
| *V* / Å^3^ | 23966.5(6) | 23957.6(3) |
| Z | 4 | 2 |
| *D_calc._* / g·cm^–3^ | 3.407 | 3.464 |
| *µ / mm^–1^* | 27.775 | 28.157 |
| *F(000)* | 21677 | 22072 |
| *Reflections measured* | 128573 | 112325 |
| *Independent Reflections* | 27251 | 22940 |
| *R_Int._* | 0.0738 | 0.0567 |
| *GOF on F^2^* | 1.055 | 1.064 |
| *Final R indices [I>2σ(I)]* | *R_1_* = 0.0456,  *wR_2_* = 0.1108 | *R_1_* = 0.0468,  *wR_2_* = 0.0949 |
| *R indices (all data)* | *R_1_* = 0.0575,  *wR_2_* = 0.1179 | *R_1_* = 0.0786,  *wR_2_* = 0.1123 |

Figure S 13. “Ball and Stick” model of europium doped BiO-NC **C-1_D_:Eu**; orange colored atoms refer to Bi atom positions doped with Eu^3+^ (Table S 6). Hydrogen atoms as well as non-coordinated molecules are omitted for clarity. The Eu^3+^ occupancies were used from Table S 6.

The M–O bond lengths in the cluster core of **C-1_D_:Eu** are 2.081(7) Å – 2.926(7) Å (undoped **C-1_D_** 2.053(11) Å – 2.981(11) Å) only slightly affected by the europium doping. Despite almost identical cell parameters the coordination environment of the NO_3_^–^ and dmso in **C-1_D_:Eu** (Table S 4) differs from the reported undoped structure **C-1_D_** as a result of the different crystallization conditions, and due to the flexible coordination ability of NO_3_^–^.

Table S 5. Summary of the Bi–O distance (*d*) ranges for different coordination modes of Bi in **C-1_D_:Eu** and **C-1_D_.**

| coordination mode | **C-1_D_:Eu** | | **C-1_D_** | |
| --- | --- | --- | --- | --- |
|  | number | *d* / Å | number | *d* / Å |
| **oxido** |  |  |  |  |
| µ_3_-1κ*O*:2κ*O*:3κ*O* | 14 | [2.083(7) – 2.531(10)] | 14 | [2.076(10) – 2.614(16)] |
| µ_4_-1κ*O*:2κ*O*:3κ*O*:4κ*O* | 30 | [2.081(7) – 2.926(7)] | 30 | [2.053(11) – 2.981(11)] |
| µ_6_-1κ*O*:2κ*O*:3κ*O*:4κ*O*:5κ*O*:6κ*O* | 1 | [2.5356(5) – 2.6081(6)] | 1 | [2.5224(6) – 2.6126(9)] |
| **nitrato** |  |  |  |  |
| bidentate | 2 | [2.668(11) – 2.894(15)] | 6 | [2.662(16) – 2.89(2)] |
| µ_2_-1κ*O*:2κ*O* | 2 | [2.848(13) – 2.936(14)] | 0 | - |
| µ_2_-1κ*O*:2κ*O*‘ | 4 | [2.842(12) – 3.046(12)] | 4 | [2.852(19) – 3.036(17)] |
| µ_2_-1κ*O*,*O*‘:2κ*O*‘ | 6* | [2.580(50) – 3.113(11)] | 4 | [2.643(14) – 3.077(18)] |
| µ_3_-1κ*O*:2κ*O*‘:3κ*O*‘ | 6* | [2.652(9) – 3.103(9)] | 2 | [2.822(13) – 2.989(14)] |
| µ_4_-1κ*O*:2κ*O*:3κ*O*:4κ*O* | 4 | [2.745(7) – 3.054(8)] | 2 | [2.765(10) – 3.032(11)] |
| µ_4_-1κ*O*:2κ*O*:3κ*O*:4κ*O*,*O*‘ | 2 | [2.791(9) – 3.089 (9)] | 2 | [2.790(12) – 3.097(15)] |
| non-coordinated | 0 | - | 2 | < 3.15 |
| **dmso** |  |  |  |  |
| µ_2_-1κ*O*:2κ*O* | 22 | [2.530(9) – 2.948(14)] | 22 | [2.707(13) – 2.972(17)] |
| µ_3_-1κ*O*:2κ*O*:3κ*O* | 4 | [2.585(8) – 3.104(10)] | 4 | [2.708(13) – 3.053(14)] |
| µ_3_-1κ*O*:2κ*O*:3κ*O*:4κ*O* | 0 | - | 2 | [2.800(18) – 3.062(19)] |
| non-coordinated | 2 | < 3.15 | 2 | < 3.15 |

*disorder

Significance test for Eu^3+^ doping in **C-1_D_:Eu**: As described above the europium occupations were compared with regard to its significance (Table S 6). Using this procedure, we determined, that in total three (half cluster) crystallographic independent positions (Figure S 14) in the BiO-NC **C-1_D_:Eu**, are doped with Eu^3+^ showing contents between 11.8(16) % (Bi15) and 6.8(18) % (Bi9). This determination, thus lead to the modified formula [Bi_37.40(34)_Eu_0.60(34)_O_145_C_56_H_168_N_24_S_28_] for **C-1_D_:Eu**.

Table S 6. Evaluation of the significance of europium doping using the difference in Eu amount of **C-1_D_:Eu** and **C-1_D_(:Eu)** (form theoretical doping of **C-1_D_** with Eu) with respect to the 3σ criteria. In the case that the 3σ value is higher than the difference in the Eu amount, it is considered to be zero.

| atom | **C-1_D_:Eu** occupancy in % | | **C-1_D_(:Eu)** occupancy in % | | difference Eu in % | Standard deviation  $\sigma=\sqrt{{\Delta(1)}^{2}+{\Delta(2)}^{2}}$ | 3σ |
| --- | --- | --- | --- | --- | --- | --- | --- |
|  | Bi | Eu | Bi | Eu |  |  |  |
| Bi2 | 0.9774(90) | 0.0227(90) | 0.9808(125) | 0.0192(125) | 0.0034 | 0.0154 | **0.046** |
| Bi5 | 0.9771(94) | 0.0229(94) | 1.00 | – | 0.0229 | 0.0163 | **0.049** |
| Bi9 | 0.9315(101) | 0.0685(101) | 0.9999(143) | 0.0011(143) | **0.0684** | 0.0175 | 0.053 |
| Bi11 | 0.8850(99) | 0.1150(99) | 1.00 | – | **0.1150** | 0.0168 | 0.050 |
| Bi14 | 0.9758(88) | 0.0242(88) | 0.9969(122) | 0.0031(122) | 0.0212 | 0.0151 | **0.045** |
| Bi15 | 0.8825(94) | 0.1175(94) | 1.00 | – | **0.1175** | 0.0160 | 0.048 |
|  |  |  |  |  | **∑ 0.3010** |  |  |

Figure S 14. Ball-and-stick” model of the cutout of BiO-NC **C-1_D_:Eu** showing the central {Bi/Eu_6_O_9_} motif including the atom labels and occupancy parameters of Bi/Eu in % as determined from the significance test.

Additionally, we examined the coordination environment of the bismuth positions partially doped by europium. In the case of BiO-NC **C-1_D_:Eu** europium occupies the inner cluster core position, which all have similar, but not identical asymmetric oxygen coordination environment. When applying the distance limit of 3.15 Å an asymmetric eight-fold coordination of Bi15/Eu15 is observed, whereas Bi9/Eu9 and Bi11/Eu11 show nine-fold coordination. A nine-fold coordination is observed considering all Bi/Eu^…^O interactions as well for Bi15/Eu15 (Figure S15), which might be described as a distorted cubic polyhedron having one additionally overcapping oxygen atom at one of the cubic planes.

Figure S 15. “Ball and Stick” model of all crystallographic different Eu coordination environments in the Eu doped BiO-NC **C-1_D_:Eu** core.

Table S 7. Summary of the different Bi/Eu^…^O distances of all europium doped positions in the BiO-NC **C-1_D_:Eu**.

| Bond | Distance (Å) | Bond | Distance (Å) | Bond | Distance (Å) |
| --- | --- | --- | --- | --- | --- |
| Bi9/Eu9–O5 | 2.346(7) | Bi11/Eu11–O12 | 2.316(7) | Bi15/Eu15–O9 | 2.276(7) |
| Bi9/Eu9–O18 | 2.377(7) | Bi11/Eu11–O5 | 2.419(7) | Bi15/Eu15–O18 | 2.357(7) |
| Bi9/Eu9–O9 | 2.421(7) | Bi11/Eu11–O18 | 2.421(7) | Bi15/Eu15–O12 | 2.390(7) |
| Bi9/Eu9–O12 | 2.425(7) | Bi11/Eu11–O9 | 2.458(7) | Bi15/Eu15–O5 | 2.426(7) |
| Bi9/Eu9–O14 | 2.5887(6) | Bi11/Eu11–O6 | 2.551(7) | Bi15/Eu15–O14 | 2.5356(5) |
| Bi9/Eu9–O7 | 2.689(7) | Bi11/Eu11–O14 | 2.6081(6) | Bi15/Eu15–O19 | 2.598(7) |
| Bi9/Eu9–O4 | 2.721(7) | Bi11/Eu11–O13 | 2.677(8) | Bi15/Eu15–O21 | 2.718(7) |
| Bi9/Eu9–O16 | 2.781(7) | Bi11/Eu11–O22 | 2.836(9) | Bi15/Eu15–O23 | 2.747(7) |
| Bi9/Eu9–O10 | 2.826(8) | Bi11/Eu11–O15 | 2.926(7) | Bi15/Eu15**^…^**O1* | 3.163(7) |

*for further information refer to bond length criteria (General remarks)

### Structures of **C-2_D_:Eu** and **C-2_D_’**:

It should be noted that cluster **C-2_D_:Eu**-**1** crystallizes in the space group *C2/c, which* differs from the previously published undoped cluster **C-2_D_.**^2^ However, we were able to crystallize the undoped BiO-NC (**C-2_D_‘**) in the space group *C2/c* and with cell parameters which are almost identical with that of BiO-NC **C-2_D_:Eu** (Table S 8). Further we synthesized **C-2_D_:Eu**-**2** starting from compound **C-2_E_:Eu-2** by recrystallisation from dmso and we were able to analyze the resulting crystals of **C-2_D_:Eu**-**2** using SC XRD resulting in same space group with similar lattice parameters compared to **C-2_D_:Eu**-**1.**

Table S 8. SC XRD crystallographic data for BiO-NCs, [Bi_38_O_45_(OMc)_24_(dmso)_4_]:Eu·4dmso·4H_2_O (**C-2_D_:Eu**-**1**), (**C-2_D_:Eu**-**2**) and [Bi_38_O_45_(OMc)_24_(dmso)_5.5_]·4dmso·4H_2_O (**C-2’**).

|  | **C-2_D_:Eu**-**1** | **C-2_D_:Eu**-**2** | **C-2_D_‘** |
| --- | --- | --- | --- |
| Empirical formula | Bi_36.81_Eu_1.19_O_105_C_112_H_176_S_8_ | Bi_37.14_Eu_0.86_O_105_C_112_H_176_S_8_ | Bi_76_O_209_C_222_H_346_S_15_ |
| *M* / g·mol^–1^ | 11332.51 | 11351.00 | 22722.35 |
| *T* / K | 100 | 100 | 100 |
| *λ* / Å | 0.71073 | 0.71073 | 0.71073 |
| Space group | *C2/c* | *C2/c* | *C2/c* |
| *a* / Å | 32.5620(12) | 32.5886(18) | 32.5693(18) |
| *b* / Å | 19.2412(7) | 19.2152(10) | 19.1972(12) |
| *c* / Å | 38.873(2) | 38.854(2) | 38.870(3) |
| *α* / ° | 90 | 90 | 90 |
| *β* / ° | 108.386(1) | 108.341(1) | 108.399(2) |
| *γ* / ° | 90 | 90 | 90 |
| *V* / Å^3^ | 23111.9(17) | 23094(2) | 23061(3) |
| Z | 4 | 4 | 2 |
| *D_calc._* / g·cm^–3^ | 3.257 | 3.265 | 3.272 |
| *µ / mm^–1^* | 28.379 | 28.515 | 29.022 |
| *F(000)* | 19785 | 19811 | 19796 |
| *Reflections measured* | 434356 | 365386 | 225798 |
| *Independent Reflections* | 28649 | 27504 | 28819 |
| *R_Int._* | 0.0799 | 0.0854 | 0.0604 |
| *GOF on F^2^* | 1.026 | 1.038 | 1.063 |
| *Final R indices [I>2σ(I)]* | *R_1_* = 0.0396,  *wR_2_* = 0.1011 | *R_1_* = 0.0386,  *wR_2_* = 0.0975 | *R_1_* = 0.0684,  *wR_2_* = 0.2073 |
| *R indices (all data)* | *R_1_* = 0.0519,  *wR_2_* = 0.1079 | *R_1_* = 0.0518,  *wR_2_* = 0.1036 | *R_1_* = 0.0773,  *wR_2_* = 0.2221 |

Please note, that a disorder of the Bi4 and Bi6 atom in the cluster core of **C-2_D_’** is observed, which is to the best of our knowledge the first example in homometallic BiO-NCs were such disordering is reported. The same disorder appears in the respective europium doped BiO-NC **C-2_D_:Eu**-**1** and **C-2_D_:Eu**-**2**. The Bi–O bond distances and coordination modes were determined for **C-2’** and exemplarily for the structure of **C-2_D_:Eu**-**1** (Table S 8). The disorder of the sulfur (namely S4, S4’) in two dmso molecules and a disorder of a methacrylate ligand (namely O1M, O2M, C1M, C2M, C2M’, C3M, C3M’ C4M, C4M’) were observed and refined. Please note: For all these structures we observed an occupational disorder of a coordinated methacrylate ligand, namely O11M, O12M and C21M–24M (occupation factor 0.7) by a dmso molecule (occupation factor 0.3, Q1;Q17;Q19). For charge neutrality at least one dmso molecule position must be thus occupied by a methacrylate ligand. While we got clear indication that such a disorder can be observed. However, we were not able to refine this disorder reliably.

Figure S 16. “Ball and Stick” model of the undoped BiO-NC **C-2_D_’**. Hydrogen atoms as well as non-coordinated molecules are omitted for clarity.

Figure S 17. “Ball and Stick” model of europium doped BiO-NC **C-2_D_:Eu**-**1** (*left*) and of BiO-NC **C-2_D_:Eu**-**2** (*right*). Hydrogen atoms as well as non-coordinated molecules are omitted for clarity.

In the case of the doped methacrylate cluster **C-2_D_:Eu**-**1**, the cluster core with M–O bond lengths in the range of 2.041(6) Å – 3.135(7) Å and a coordination environment with 14 µ_3_-1κ*O*:2κ*O*:3κ*O*, 30 µ_4_-1κ*O*:2κ*O*:3κ*O*:4κ*O* and one µ_6_-1κ*O*:2κ*O*:3κ*O*:4κ*O*:5κ*O*:6κ*O* modes differs only slightly from the undoped methacrylate cluster **C-2_D_’** with Bi–O distances 2.022(10) Å – 3.079(11) Å and core connectivity with 16 µ_3_-1κ*O*:2κ*O*:3κ*O*, 28 µ_4_-1κ*O*:2κ*O*:3κ*O*:4κ*O* and one µ_6_-1κ*O*:2κ*O*:3κ*O*:4κ*O*:5κ*O*:6κ*O* (Table S 9). The coordination modes of the ligands are identical for the europium doped and undoped cluster **C-2_D_:Eu**-**1**, **C-2_D_’** (Table S 9), showing only minor differences in their M–O bond distances.

Table S 9. Summary of the M–O distance (*d*) ranges for different coordination modes of M in **C-2_D_:Eu-2** and **C-2_D_’.**

| coordination mode | **C-2_D_:Eu**-**2** | | **C-2_D_‘** | |
| --- | --- | --- | --- | --- |
|  | number | *d* / Å | number | *d* / Å |
| **oxido** |  |  |  |  |
| µ_3_-1κ*O*:2κ*O*:3κ*O* | 14 | [2.092(7) – 2.523(8)] | 16 | [2.077(9) – 2.575(15)] |
| µ_4_-1κ*O*:2κ*O*:3κ*O*:4κ*O* | 30 | [2.041(6) – 3.135(7)] | 28 | [2.022(10) – 3.079(11)] |
| µ_6_-1κ*O*:2κ*O*:3κ*O*:4κ*O*:5κ*O*:6κ*O* | 1 | [2.450(6) – 2.904(8)] | 1 | [2.064(9) – 2.707(9) |
| **dmso** |  |  |  |  |
| µ_2_-1κ*O*:2κ*O* | 6 | [2.694(8) – 2.915(8)] | 6 | [2.687(10) – 2.919(11)] |
| µ_2_-1κ*O*:2κ*O*:3k*O* | 2 | [2.639(17) – 3.128(15)] | 2 | [2.64(2) – 3.13(2) |
| **methacrylato** |  |  |  |  |
| µ_2_-1κ*O*:2κ*O*‘ | 2 | [2.631(9) – 2.799(10)] | 2 | [2.615(11) – 2.781(14)] |
| µ_2_-1κ*O*,*O*‘:2κ*O*‘ | 6 | [2.478(13) – 2.971(16)] | 6 | [2.493(15) – 2.942(18)] |
| µ_3_-1κ*O*:2κ*O*‘:3κ*O*‘ | 6 | [2.572(8) – 3.135(13)] | 6 | [2.570(10) – 3.112(13)] |
| µ_3_-1κ*O*:2κ*O*:3κ*O*,*O*‘ | 4 | [2.428(13) – 3.108(13)] | 4 | [2.374(17) – 3.100(16)] |
| µ_4_-1κ*O*:2κ*O*:3κ*O*‘:4κ*O*‘ | 6 | [2.599(8) – 2.815(10)] | 6 | [2.597(11) – 2.825(13)] |
| **aqua/hydroxido** |  |  |  |  |
| 1κ*O* | 4 | [2.805(8) – 2.863(9)] | 4 | [2.800(12) – 2.846(13)] |

Figure S 18. Section of the crystal structure of **C-2_D_:Eu**-**2** to visualise the nearly hexagonal close packing of the cluster molecules with highlighted lattice plains [000], [100], [200]. Hydrogen and carbon atoms as well as non-coordinated molecules are omitted for clarity.

Significance Test for Eu^3+^ doping in **C-2_D_:Eu**: According to the introduced procedure; see above and references^6-7^, **C-2_D_:Eu** does not show significance in Eu-doping by means of SC XRD studies after the as described correction procedure (Table S 10 **C-2_D_:Eu-1**, Table S 11 **C-2_D_:Eu-2**). Thus, the crystals of **C-2_E_:Eu**-**2** (positive significance test) were dissolved in dmso and were crystallized to give **C-2_D_:Eu**-**2**, showing almost identical cell parameters as **C-2_D_:Eu**-**1**. These independently obtained crystals did confirm the results as obtained for **C-2_D_:Eu**.

On the condition that there is no disassembly and reassembly of the cluster core during the recrystallization process, which was proven previously,^17^ the Eu amount is not expected to be changed. In addition, europium is detected by using ESI-MS, ICP-OES and PL measurements for both crystalline materials, wherefore we assume that in the case of cluster **C-2_D_:Eu (C-2_D_:Eu**-**2**), the bismuth positions are indeed partially occupied by europium, Although the original dataset indicates substitutional disorder and additional experiments verify the doping, the 3σ criterion does not allow for any assignment of the Eu positions.

Table S 10. Evaluation of the significant amount of europium using the difference in Eu amount of **C-2_D_:Eu-1** and **C-2_D_’(:Eu)-1** (form theoretical doping of **C-2_D_’** with Eu) with respect to the 3σ criteria. In the case that the 3σ value is higher than the difference in the Eu amount, it is considered to be zero.

| atom | **C-2_D_:Eu**-**1** occupancy in % | | **C-2_D_’(:Eu)-1** occupancy in % | | difference Eu in % | Standard deviation  $\sigma=\sqrt{{\Delta(1)}^{2}+{\Delta(2)}^{2}}$ | 3σ |
| --- | --- | --- | --- | --- | --- | --- | --- |
|  | Bi | Eu | Bi | Eu | Δ(Eu) |  |  |
| Bi2 | 0.9696(89) | 0.0304(89) | 0.9855(129) | 0.0145(129) | 0.0159 | 0.0157 | **0.047** |
| Bi3 | 0.9662(89) | 0.0338(89) | 0.9618(129) | 0.0382(129) | -0.0044 | 0.0156 | **0.047** |
| Bi4 | 0.4153(705) | 0.0447(705) | 0.4560(699) | 0.0000(699) | 0.0447 | 0.1986 | **0.596** |
| Bi4‘ | 0.4441(606) | 0.0959(606) | 0.4080(603) | 0.1320(603) | -0.0361 | 0.1709 | **0.513** |
| Bi5 | 0.9641(88) | 0.0359(88) | 0.9787(123) | 0.0213(129) | 0.0146 | 0.0156 | **0.047** |
| Bi6 | 0.6300 | 0.0000 | 0.6300 | 0.0000 | - | - | **-** |
| Bi6‘ | 0.3224(241) | 0.0478(241) | 0.3679(352) | 0.0023(352) | 0.0455 | 0.0426 | **0.128** |
| Bi7 | 0.9528(90) | 0.0472(90) | 1.0000(131) | 0.0000(131) | 0.0472 | 0.0158 | **0.048** |
| Bi9 | 0.9252(93) | 0.0748(93) | 0.9492(131) | 0.0508(131) | 0.0240 | 0.0161 | **0.048** |
| Bi10 | 0.9554(90) | 0.0446(90) | 0.9825(125) | 0.0175(125) | 0.0271 | 0.0154 | **0.046** |
| Bi11 | 0.9429(83) | 0.05711(83) | 0.9055(115) | 0.0945(115) | -0.0374 | 0.0142 | **0.043** |
| Bi13 | 0.9769(89) | 0.0231(89) | 0.9807(129) | 0.0193(129) | 0.0038 | 0.0156 | **0.047** |
| Bi15 | 0.9966(89) | 0.0034(89) | 0.9655(128) | 0.0345(128) | -0.0311 | 0.0155 | **0.047** |
| Bi16 | 0.9500(90) | 0.0500(90) | 0.9618(130) | 0.0383(130) | 0.0118 | 0.0158 | **0.047** |
| Bi17 | 0.9943(89) | 0.0057(89) | 1.0000(134) | 0.0000(134) | 0.0057 | 0.0161 | **0.048** |
|  |  |  |  |  | **∑ 0.000** |  |  |

Table S 11. Evaluation of the significant amount of europium using the difference in Eu amount of **C-2_D_:Eu-2** and **C-2_D_’(:Eu)-2** (form theoretical doping of **C-2_D_’** with Eu) with respect to the 3σ criteria. In the case that the 3σ value is higher than the difference in the Eu amount, it is considered to be zero

| atom | **C-2_D_:Eu**-**2** occupancy in % | | **C-2_D_’(:Eu)**-**2** occupancy in % | | difference Eu in % | Standard deviation  $\sigma=\sqrt{{\Delta(1)}^{2}+{\Delta(2)}^{2}}$ | 3σ |
| --- | --- | --- | --- | --- | --- | --- | --- |
|  | Bi | Eu | Bi | Eu | Δ(Eu) |  |  |
| Bi2 | 0.9871(88) | 0.0129(88) | 0.9965(145) | 0.0035(145) | 0.0094 | 0.0170 | **0.051** |
| Bi3 | 0.9830(88) | 0.0170(88) | 0.9161(144) | 0.0839(144) | -0.0669 | 0.0168 | **0.050** |
| Bi4 | 0.3999 | 0.0000 | 0.3999 | 0.0000 | 0.0000 | - | **-** |
| Bi4‘ | 0.4610(171) | 0.1390(171) | 0.5063(282) | 0.0937(282) | 0.0453 | 0.0330 | **0.099** |
| Bi5 | 0.9804(87) | 0.0196(87) | 0.9882(144) | 0.0118(144) | 0.0077 | 0.0168 | **0.050** |
| Bi6 | 0.6700 | 0.0000 | 0.6700 | 0.0000 | 0.0000 | - | **-** |
| Bi6‘ | 0.3109(266) | 0.0191(266) | 0.3094(444) | 0.0206(444) | -0.0015 | 0.0518 | **0.155** |
| Bi7 | 0.9830(88) | 0.0170(88) | 1.0000(147) | 0.0000(147) | 0.0170 | 0.0171 | **0.051** |
| Bi9 | 0.9228(91) | 0.0772(91) | 0.9314(146) | 0.0686(146) | -0.0086 | 0.0172 | **0.052** |
| Bi10 | 0.9510(89) | 0.0490(89) | 0.9226(141) | 0.0774(141) | -0.0285 | 0.0166 | **0.050** |
| Bi11 | 0.9668(82) | 0.0332(82) | 0.9089(131) | 0.0911(131) | -0.0578 | 0.0155 | **0.046** |
| Bi15 | 0.9872(87) | 0.0128(87) | 1.0000(143) | 0.0000(143) | 0.0128 | 0.0167 | **0.050** |
| Bi16 | 0.9649(89) | 0.0351(89) | 0.9497(147) | 0.0503(147) | -0.0152 | 0.0172 | **0.052** |
|  |  |  |  |  | **∑ 0.000** |  |  |

### Structure of **C-2_E_:Eu**:

It should be noted that cluster **C-2_E_:Eu** crystallizes in the same space group with similar unit cell parameters as compared to the previously published undoped cluster **C-2_E_**.^4^ However, in order to evaluate the europium doping in structure **C-2_E_:Eu** we performed a new SC SXRD measurement of the undoped BiO-NC (**C-2_E_’**) using the identical diffractometer and data integration software as used for **C-2_E_:Eu**. Further, we aimed to use for both independent measurements crystals of the same size and aimed to measure with related redundancies in order to ensure a uniform data identity. For the structural comparison with the doped BiO-NC **C-2_E_:Eu** herein we use the new refined structure of the undoped cluster (**C-2_E_’**). (Table S 12). Furthermore, in the case of BiO-NC **C-2_E_:Eu**, we carried out a second SC XRD analysis on a second crystal from a different synthesis approach in order to compare both measurements with regard to europium doping. The results for **C-2_E_:Eu**-**1** and **C-2_E_:Eu**-**2** are summarized in Table S 12, while the determination of the europium content for the structures is summarized in Table S 14 (**C-2_E_:Eu**-**1**) and Table S 16 (**C-2_E_:Eu**-**2**).

Table S 12. SC XRD crystallographic data for the different europium doped BiO-NCs, [Bi_38_O_45_(OMc)_24_(EtOH)_10_]:Eu·4EtOH (**C-2_E_:Eu**-**1**), [Bi_38_O_45_(OMc)_24_(EtOH)_10_]:Eu·4EtOH (**C-2_E_:Eu**-**2**), [Bi_38_O_45_(OMc)_24_(EtOH)_14_] (**C-2_E_’**).

|  | **C-2_E_:Eu**-**1** | **C-2_E_:Eu**-**2** | **C-2_E_’** |
| --- | --- | --- | --- |
| Empirical formula | Bi_35.23_Eu_2.77_O_107_C_124_H_202_ | Bi_35.73_Eu_2.27_O_107_C_124_H_202_ | Bi_38_O_107_C_124_H_202_ |
| *M* / g·mol^–1^ | 11188.25 | 11216.59 | 11346.08 |
| *T* / K | 100 | 100 | 100(2) |
| *λ* / Å | 0.71073 | 0.71073 | 0.71073 |
| *Space group* | *P*$\bar{1}$ | *P*$\bar{1}$ | *P*$\bar{1}$ |
| *a* / Å | 16.991(3) | 17.020(3) | 16.9833(11) |
| *b* / Å | 18.989(4) | 18.987(3) | 18.9862(11) |
| *c* / Å | 20.198(4) | 20.245(4) | 20.1839(13) |
| *α* / ° | 111.195(6) | 111.178(6) | 111.157(2) |
| *β* / ° | 111.404(6) | 111.410(6) | 111.443(2) |
| *γ* / ° | 100.632(7) | 100.643(6) | 100.725(2) |
| *V* / Å^3^ | 5267.6(17) | 5288.3(16) | 5256.6(6) |
| Z | 1 | 1 | 1 |
| *D_calc._* / g·cm^–3^ | 3.527 | 3.522 | 3.584 |
| *µ / mm^–1^* | 30.200 | 30.348 | 31.759 |
| *F(000)* | 4900.6 | 4911 | 4956 |
| *Reflections measured* | 422604 | 283110 | 601223 |
| *Independent Reflections* | 25443 | 23040 | 22908 |
| *R_Int._* | 0.0568 | 0.0854 | 0.0599 |
| *GOF on F^2^* | 1.070 | 1.064 | 1.112 |
| *Final R indices [I>2σ(I)]* | *R1* = 0.0488,  *wR2* = 0.1321 | *R1* = 0.0437,  *wR2* = 0.1041 | *R1* = 0.0234,  *wR2* = 0.0537 |
| *R indices (all data)* | *R1* = 0.0527,  *wR2* = 0.1361 | *R1* = 0.0573,  *wR2* = 0.1089 | *R1* = 0.0260,  *wR2* = 0.0546 |

In the undoped structure **C-2_E_’**, disorder of Bi positions was not observed and all Bi atoms are refined using the constrains EXYZ and EADP. Please note, that in contrast to the refinement of the crystallographic data of BiO-NC **C-2_E_:Eu-1** six Bi/Eu positions and in **C-2_E_:Eu-2** seven Bi/Eu positions were fixed using the constrains EXYZ and EADP, whereas the other positions are refined freely leading to separate Bi and Eu positions. This was necessary, as high unrefined electron density peaks near certain Bi positions were observed. While this is quite often the case for BiO-NCs, especially when crystallized from dmso solution by allowing solvents with higher vapor pressure (*e.g.* acetone or THF) to diffuse into them, it was not observed for crystals of BiO-NCs crystallized from EtOH solution. According to our so far unpublished experiences of this dmso effect, the presence of acetone, THF or any other solvent with low boiling point upon crystal growth by the above mention diffusion method lead to a very rapid rupturing of the single crystals in the moment they are taken from *e.g.* dmso/solvent solution. This is due to the packing solvents showing high vapor pressure, thus leaving the crystals. This usually leads to domain formation, but was never observed for single crystals obtained upon slow evaporation of dmso as alternative crystallization method (where successful). It was thus tried to refine the Eu/Bi position independently without EXYZ and EADP and was successful for some positions. Due to different radii of Bi versus Eu it is understood that an independent refinement of the Eu and Bi atoms occupying related positions in doped BiO-NCs describes the situation better compared to constraining them to almost identical positions.

Figure S 19. “Ball and Stick” model of undoped BiO-NC **C-2_E_’**. Hydrogen atoms as well as non-coordinated molecules are omitted for clarity.

Figure S 20. “Ball and Stick” model of europium doped BiO-NC **C-2_E_:Eu**-**1** (*left*) and **C-2_E_:Eu**-**2** (*right*), orange colored atoms refer to Bi atom positions doped with Eu. Hydrogen atoms as well as non-coordinated molecules are omitted for clarity.

The Bi–O bond lengths and the coordination modes were determined for the undoped cluster **C-2_E_'** and exemplary, in the europium doped cluster **C-2_E_:Eu**-**1**. When compared to **C-2_E_'**, the separation of Bi/Eu positions in **C-2_E_:Eu** leads to slightly different bond distances in the cluster core as well as in the ligand shell, where further different coordination modes were observed. The respective coordination modes and bond distances for the cluster core as well as in the ligand shell were determined twice. One time using only the Bi atoms (**C-2_E_:Eu**-**1 (Bi)**) separate refined positions were used and one time the respective Eu atoms (**C-2_E_:Eu**-**1 (Eu)**) were used according to Table S 14. In our opinion the combination of both variants completely describes the coordination of BiO-NC **C-2_E_:Eu**-**1** best. The resulting coordination modes and bond lengths are summarized in Table S13.

Table S 13. Summary of the M–O distance (*d*) ranges for different coordination modes of M in **C-2_E_:Eu**-**1 (Bi)**, **C-2_E_:Eu**-**1 (Eu)** and **C-2_E_’.**

| coordination mode | **C-2_E_:Eu**-**1 (Bi)** | | **C-2_E_:Eu**-**1 (Eu)** | |
| --- | --- | --- | --- | --- |
|  | number | *d* / Å | number | *d /* Å |
| **oxido** |  |  |  |  |
| µ_3_-1κ*O*:2κ*O*:3κ*O* | 16 | [2.101(6) – 2.417(7)] | 14 | [2.041(17) – 2.587(19)] |
| µ_4_-1κ*O*:2κ*O*:3κ*O*:4κ*O* | 28 | [2.062(6) – 3.080(7)] | 30 | [2.079(6) – 3.114(11)] |
| µ_6_-1κ*O*:2κ*O*:3κ*O*:4κ*O*:5κ*O*:6κ*O* | 1 | [2.404(2) – 2.848(3)] | 1 | [2.594(6) – 2.698(9)] |
| **EtOH** |  |  |  |  |
| 1κ*O* | 8 | [2.747(8) – 2.971(10)] | 6 | [2.747(8) – 2.971(10)] |
| µ_2_-1κ*O*:2κ*O* | 6 | [2.727(8) – 3.063(9)] | 4 | [2.56(6) – 3.063(9)] |
| non-coordinated |  |  | 2 | >3.15 |
| **methacrylato** |  |  |  |  |
| µ_2_-1κ*O*:2κ*O* | 6 | [2.485(7) – 2.820(8)] | 6 | [2.485(7) – 3.13(6)] |
| µ_2_-1κ*O*,*O*‘:2κ*O*‘ | 4 | [2.382(9) – 3.020(9)] | 2 | [2.420(7) – 2.874(7)] |
| µ_3_-1κ*O*:2κ*O*‘:3κ*O*‘ | 4 | [2.639(11) – 3.066(9)] | 4 | [2.22(6) – 3.066(9)] |
| µ_3_-1κ*O*:2κ*O*:3κ*O*,*O*‘ | 0 | - | 2 | [2.382(9) – 2.91(2)] |
| µ_3_-1κ*O*:2κ*O*:2κ*O*’:3κ*O*’ | 4 | [2.410(7) – 3.142(8)] | 4 | [2.410(7) – 3.142(8)] |
| µ_4_-1κ*O*:2κ*O*:3κ*O*‘:4κ*O*‘ | 4 | [2.563(7) – 2.907(8)] | 4 | [2.563(7) – 3.15(5)] |
| µ_4_-1κ*O*:2κ*O*:3κ*O*:3κ*O*‘:4κ*O*‘ | 2 | [2.722(7) – 3.063(8)] | 2 | [2.722(7) – 3.063(8)] |

| coordination mode | **C-2_E_*‘*** | |
| --- | --- | --- |
|  | number | *d / Å* |
| **oxido** |  |  |
| µ_3_-1κ*O*:2κ*O*:3κ*O* | 16 | [2.091(5) – 2.417(5)] |
| µ_4_-1κ*O*:2κ*O*:3κ*O*:4κ*O* | 28 | [2.065(5) – 3.055(5)] |
| µ_6_-1κ*O*:2κ*O*:3κ*O*:4κ*O*:5κ*O*:6κ*O* | 1 | [2.4281(3) – 2.8133(3)] |
| **EtOH** |  |  |
| 1κ*O* | 8 | [2.737(6) – 2.977(7)] |
| µ_2_-1κ*O*:2κ*O* | 6 | [2.739(6) – 3.072(6)] |
| non-coordinated |  | >3.15 |
| **methacrylato** |  |  |
| µ_2_-1κ*O*:2κ*O* | 6 | [2.489(6) – 2.804(6)] |
| µ_2_-1κ*O*,*O*‘:2κ*O*‘ | 4 | [2.386(8) – 3.021(7)] |
| µ_3_-1κ*O*:2κ*O*‘:3κ*O*‘ | 4 | [2.625(8) – 3.043(6)] |
| µ_3_-1κ*O*:2κ*O*:3κ*O*,*O*‘ | 0 | - |
| µ_3_-1κ*O*:2κ*O*:2κ*O*’:3κ*O*’ | 4 | [2.398(5) – 3.148(6)] |
| µ_4_-1κ*O*:2κ*O*:3κ*O*‘:4κ*O*‘ | 4 | [2.556(6) – 2.899(6)] |
| µ_4_-1κ*O*:2κ*O*:3κ*O*:3κ*O*‘:4κ*O*‘ | 2 | [2.695(6) – 3.088(6)] |

The resulting distances are in good agreement with respective values in the measurement of the undoped BiO-NC **C-2_E_'**. Slight deviations in the M–O distances of the cluster core are most likely caused by the determination of the bond distance at **C-2_E_:Eu** with separate refined Bi/Eu atoms. The coordination environment of **C-2_E_:Eu** and **C-2_E_'** (Table S 13) in the ligand sphere are identical when considering only the bismuth positions in **C-2_E_:Eu**-**1(Bi)**. When coordination environment of the europium doped positions in **C-2_E_:Eu**-**1(Eu)** is determined, the coordination modes of two oxygen atoms in the core changed from µ_3_-1κ*O*:2κ*O*:3κ*O* to µ_4_-1κ*O*:2κ*O*:3κ*O*:4κ*O* and the coordination of two methacrylate change from µ_2_-1κ*O*,*O*‘:2κ*O*‘ to µ_3_-1κ*O*:2κ*O*:3κ*O*,*O*‘ compared to **C-2_E_'**. The range of the respective bond length is only slightly affected by the use of the different atoms.

Significance test for Eu^3+^ doping in **C-2_E_:Eu**: As described above the europium occupations were compared with regard to its significance (Table S 14). This determination, thus lead to modified formula of [Bi_36.36(50)_Eu_1.64(50)_O_107_C_124_H_202_] for **C-2_E_:Eu**-**1** and [Bi_37.16(46)_Eu_0.84(46)_O_107_C_124_H_202_] for **C-2_E_:Eu**-**2** (Table S 16).

Table S 14. Evaluation of the significant amount of europium using the difference in Eu amount of **C-2_E_:Eu-1** and **C-2_E_’(:Eu)-1** (form theoretical doping of **C-2_E_’** with Eu) with respect to the 3σ criteria. In the case that the 3σ value is higher than the difference in the Eu amount, it is considered to be zero.

| atom | **C-2_E_:Eu**-**1** occupancy in % | | **C-2_E_’(:Eu)-1** occupancy in % | | difference Eu in % | Standard deviation  $\sigma=\sqrt{{\Delta(1)}^{2}+{\Delta(2)}^{2}}$ | 3σ |
| --- | --- | --- | --- | --- | --- | --- | --- |
|  | Bi | Eu | Bi | Eu | Δ(Eu) |  |  |
| Bi1 | 0.9895(19) | 0.0105(19) | 0.9945(07) | 0.0056(07) | 0.0049 | 0.0020 | **0.0060** |
| Bi2 | 0.9855(97) | 0.0145(97) | 0.9657(56) | 0.0343(56) | -0.0198 | 0.0112 | **0.0335** |
| Bi3 | 0.8895(92) | 0.1105(92) | 0.9568(55) | 0.0432(55) | **0.0673** | 0.0107 | 0.0322 |
| Bi4 | 0.7768(96) | 0.2232(96) | 0.9461(52) | 0.0539(52) | **0.1693** | 0.0109 | 0.0326 |
| Bi5 | 0.9862(38) | 0.0138(38) | 0.9918(17) | 0.0082(17) | 0.0056 | 0.0041 | **0.0124** |
| Bi6 | 0.6852(97) | 0.3148(97) | 0.9510(54) | 0.0490(54) | **0.2658** | 0.0111 | 0.0334 |
| Bi7 | 0.9767(93) | 0.0233(93) | 0.9742(56) | 0.0258(56) | -0.0025 | 0.0109 | **0.0326** |
| Bi8 | 0.9795(52) | 0.0205(52) | 0.9707(57) | 0.0293(57) | -0.0088 | 0.0078 | **0.0233** |
| Bi9 | 0.9008(92) | 0.0993(92) | 0.9483(55) | 0.0517(55) | **0.0475** | 0.0107 | 0.0320 |
| Bi10 | 0.9019(94) | 0.0981(94) | 0.9578(56) | 0.0422(56) | **0.0559** | 0.0110 | 0.0329 |
| Bi11 | 0.8946(93) | 0.1054(93) | 0.9568(57) | 0.0432(57) | **0.0622** | 0.0109 | 0.0327 |
| Bi12 | 0.9557(91) | 0.0443(91) | 0.9662(56) | 0.0338(56) | 0.0105 | 0.0107 | **0.0320** |
| Bi13 | 0.8186(94) | 0.1814(94) | 0.9690(56) | 0.0310(56) | **0.1504** | 0.0109 | 0.0328 |
| Bi14 | 0.9517(91) | 0.0483(91) | 0.9672(55) | 0.0328(55) | 0.0155 | 0.0106 | **0.0319** |
| Bi15 | 0.9701(95) | 0.0299(95) | 0.9595(56) | 0.0405(56) | -0.0105 | 0.0110 | **0.0331** |
| Bi16 | 0.9907(12) | 0.0093(12) | 0.9945(07) | 0.0055(07) | 0.0034 | 0.0014 | **0.0042** |
| Bi17 | 0.9860(21) | 0.0140(21) | 0.9685(56) | 0.0315(56) | -0.0176 | 0.0060 | **0.0179** |
| Bi18 | 0.9893(94) | 0.0107(94) | 0.9772(56) | 0.0229(56) | -0.0122 | 0.0109 | **0.0327** |
| Bi19 | 0.9874(92) | 0.0126(92) | 0.9703(56) | 0.0297(56) | -0.0171 | 0.0107 | **0.0322** |
|  |  |  |  |  | **∑ 0.8184** |  |  |

Figure S 21. Ball-and-stick” model of the cutout of BiO-NC **C-2_E_:Eu**-**1** showing the central {Bi/Eu_6_O_9_} motif with the adjusted Bi3/Eu3, Bi9/Eu9, Bi10/Eu10, Bi11/Eu11 atoms including the occupancy parameters of Bi/Eu in % determined from the significance test.

Figure S 22. “Ball and Stick” of all crystallographic different Eu coordination environments in the Eu^3+^ doped BiO-NC **C-2_E_:Eu**-**1** core. Eu4, Eu6, and Eu13 belong to the inner [Bi/Eu_6_O_9_] octahedron and Eu3, Eu9, Eu10 and Eu11 are adjusted positions.

In the case of BiO-NC **C-2_E_:Eu**-**1** europium occupies not only the inner cluster core positions, but as well positions connected via the µ_3_-briding oxygen atoms of the [Bi/Eu_6_O_9_]-octahedron. The inner octahedral Eu positions have similar, but not identical asymmetric oxygen coordination environment as described for **C-1_D_:Eu**. When considering all oxygen atoms interacting within the distance lower than the sum of the van der Waals radii a nine-fold coordination is observed only for Eu9 and Eu10, whereas Eu11 shows ten-fold coordination, Eu3 and Eu6 eleven-fold coordination and Eu13 twelve-fold coordination (Figure S 22). Therefore, all Eu atoms in **C-2_E_:Eu**-**1** show different and strongly asymmetric coordination geometries, which is stronger compared to **C-1_D_:Eu**. Further all outer Eu positions show three different ranges of Eu–O distance regimes: short ones within ≈2.0 Å – 2.25 Å, middle ones in the range of ≈2.4 Å – 2.65 Å and longer ones <2.75 Å.

Table S 15. Summary of the different Bi/Eu^…^O distances of all europium doped positions in the BiO-NC **C-2_E_:Eu**-**1**.

| **Bond** | **Distance (Å)** | **Bond** | **Distance (Å)** | **Bond** | **Distance (Å)** |
| --- | --- | --- | --- | --- | --- |
| Eu3–O5 | 2.07(2) | Eu4–O2 | 2.406(10) | Eu6–O1 | 2.353(10) |
| Eu3–O11 | 2.141(19) | Eu4–O1 | 2.418(10) | Eu6–O8 | 2.360(9) |
| Eu3–O18 | 2.155(17) | Eu4–O11 | 2.475(10) | Eu6–O2 | 2.461(9) |
| Eu3–O13 | 2.424(18) | Eu4–O8 | 2.489(10) | Eu6–O9 | 2.515(9) |
| Eu3–O31 | 2.76(2) | Eu4–O36 | 2.508(10) | Eu6–O11 | 2.521(9) |
| Eu3–O16 | 2.91(2) | Eu4–O41 | 2.661(10) | Eu6–O32 | 2.594(6) |
| Eu3–O10 | 2.99(2) | Eu4–O32 | 2.688(7) | Eu6–O39 | 2.606(9) |
| Eu3^…^O19* | 3.476(18) | Eu4–O7 | 2.730(10) | Eu6–O6 | 2.633(9) |
| Eu3^…^O24* | 3.557(19) | Eu4**^…^**O13* | 3.320(10) | Eu6–O18 | 2.803(10) |
| Eu3^…^O30* | 3.72(2) | Eu4**^…^**O29* | 3.321(11) | Eu6**^…^**O5* | 3.724(10) |
| Eu3^…^O26* | 3.726(17) | Eu4**^…^**O5* | 3.676(10) | Eu6**^…^**O24* | 3.839(10) |
|  |  | Eu4**^…^**O26* | 3.800(10) |  |  |
|  |  |  |  |  |  |
| **Bond** | **Distance (Å)** | **Bond** | **Distance (Å)** | **Bond** | **Distance (Å)** |
| Eu9–O2 | 2.085(17) | Eu10–O1 | 2.15(3) | Eu11–O12 | 2.041(17) |
| Eu9–O38 | 2.147(17) | Eu10–O4 | 2.16(3) | Eu11–O8 | 2.146(19) |
| Eu9–O24 | 2.292(17) | Eu10–O7 | 2.37(3) | Eu11–O3 | 2.226(19) |
| Eu9–O41 | 2.557(17) | Eu10–O26 | 2.42(3) | Eu11–O39 | 2.54(2) |
| Eu9–O6 | 2.570(17) | Eu10–O9 | 2.61(3) | Eu11–O29 | 2.587(19) |
| Eu9**^…^**O29* | 3.176(17) | Eu10–O33 | 2.66(2) | Eu11–O44 | 3.09(2) |
| Eu9**^…^**O47* | 3.297(18) | Eu10–O42 | 2.88(3) | Eu11–O36 | 3.108(17) |
| Eu9**^…^**O34* | 3.328(17) | Eu10**^…^**O43* | 3.48(3) | Eu11**^…^**O51* | 3.17(2) |
| Eu9**^…^**O25* | 3.340(17) | Eu10**^…^**O49* | 3.85(3) | Eu11**^…^**O45* | 3.54(2) |
|  |  |  |  | Eu11**^…^**O23* | 3.69(2) |
| **Bond** | **Distance (Å)** |  |  |  |  |
| Eu13–O11 | 2.383(11) |  |  |  |  |
| Eu13–O2 | 2.411(11) |  |  |  |  |
| Eu13–O10 | 2.436(11) |  |  |  |  |
| Eu13–O8 | 2.487(11) |  |  |  |  |
| Eu13–O1 | 2.496(11) |  |  |  |  |
| Eu13–O32 | 2.698(9) |  |  |  |  |
| Eu13–O33 | 2.718(12) |  |  |  |  |
| Eu13–O38 | 2.903(11) |  |  |  |  |
| Eu13–O3 | 3.114(11) |  |  |  |  |
| Eu13**^…^**O26* | 3.428(12) |  |  |  |  |
| Eu13**^…^**O24* | 3.695(11) |  |  |  |  |
| Eu13**^…^**O29* | 3.695(11) |  |  |  |  |

*for further information see bond length criteria (General remarks)

Table S 16. Evaluation of the significant amount of europium using the difference in Eu amount of **C-2_E_:Eu-2** and **C-2_E_^’^(:Eu)-2** (form theoretical doping of **C-2_E_’** with Eu) with respect to the 3σ criteria. In the case that the 3σ value is higher than the difference in the Eu amount, it is considered to be zero.

| atom | **C-2_E_:Eu**-**2** occupancy in % | | **C-2_E_’(:Eu)**-**2** occupancy in % | | difference Eu in % | Standard deviation  $\sigma=\sqrt{{\Delta(1)}^{2}+{\Delta(2)}^{2}}$ | 3σ |
| --- | --- | --- | --- | --- | --- | --- | --- |
|  | Bi | Eu | Bi | Eu | Δ(Eu) |  |  |
| Bi1 | 0.9951(14) | 0.0049(14) | 0.9959(07) | 0.0041(07) | 0.0008 | 0.0016 | **0.0047** |
| Bi2 | 0.9700(115) | 0.0301(115) | 0.9674(56) | 0.0326(56) | -0.0026 | 0.0128 | **0.0383** |
| Bi3 | 0.9337(115) | 0.0663(115) | 0.9605(55) | 0.0395(55) | 0.0268 | 0.0127 | **0.0382** |
| Bi4 | 0.8322(115) | 0.1678(115) | 0.9369(56) | 0.0631(56) | **0.1047** | 0.0128 | 0.0383 |
| Bi5 | 0.9789(67) | 0.0211(67) | 0.9920(18) | 0.0080(18) | 0.0131 | 0.0070 | **0.0209** |
| Bi6 | 0.7703(118) | 0.2297(118) | 0.9494(55) | 0.0506(55) | **0.1791** | 0.0130 | 0.0390 |
| Bi7 | 0.9897(114) | 0.0103(114) | 0.9766(56) | 0.0234(56) | -0.0131 | 0.0127 | **0.0381** |
| Bi8 | 0.9604(118) | 0.0396(118) | 0.9711(58) | 0.0289(58) | 0.0107 | 0.0131 | **0.0393** |
| Bi9 | 0.9375(113) | 0.0625(113) | 0.9510(55) | 0.0490(55) | 0.0135 | 0.0126 | **0.0378** |
| Bi10 | 0.9239(114) | 0.0761(114) | 0.9598(56) | 0.0402(56) | 0.0359 | 0.0127 | **0.0382** |
| Bi11 | 0.9267(114) | 0.0733(114) | 0.9612(58) | 0.0388(58) | 0.0345 | 0.0128 | **0.0384** |
| Bi12 | 0.9303(113) | 0.0697(113) | 0.9718(56) | 0.0283(56) | **0.0415** | 0.0126 | 0.0378 |
| Bi13 | 0.8778(115) | 0.1222(115) | 0.9705(54) | 0.0295(54) | **0.0926** | 0.0127 | 0.0382 |
| Bi14 | 0.9556(112) | 0.0444(112) | 0.9721(55) | 0.0279(55) | 0.0165 | 0.0125 | **0.0375** |
| Bi15 | 0.9500(115) | 0.0500(115) | 0.9634(56) | 0.0366(56) | 0.0134 | 0.0128 | **0.0384** |
| Bi16 | 0.9884(19) | 0.0116(19) | 0.9940(08) | 0.0060(08) | 0.0056 | 0.0020 | **0.0061** |
| Bi17 | 0.9912(17) | 0.0088(17) | 0.9943(08) | 0.0057(08) | 0.0031 | 0.0018 | **0.0054** |
| Bi18 | 0.9623(114) | 0.0378(114) | 0.9803(56) | 0.0197(56) | 0.0180 | 0.0127 | **0.0381** |
| Bi19 | 0.9902(114) | 0.0098(114) | 0.9744(56) | 0.0256(56) | -0.0158 | 0.0127 | **0.0380** |
|  |  |  |  |  | **∑ 0.4179** |  |  |

Figure S 23. Ball-and-stick” model of the cutout of BiO-NC **C-2_E_:Eu**-**2** showing the central {Bi/Eu_6_O_9_} motif with the adjusted Bi12/Eu12 atoms including the occupancy parameters of Bi/Eu in % determined from the significance test.

Table S 17. Summary of the europium content values and the Eu/Bi ratios of the BiO-NCs **C-1_D_:Eu, C-2_D_:Eu** and **C-2_E_:Eu** as determined by SC XRD and as corrected using the significance test.

| **BiO-NC** | **Eu [ω%]** | | **Eu/Bi** | |
| --- | --- | --- | --- | --- |
|  | **SC XRD**  **(corrected)** | **SC XRD (measured)** | **SC XRD**  **(corrected)** | **SC XRD (measured)** |
| **C-1_D_:Eu** | 0.74(6) | 0.91 | 0.012 | 0.014 |
| **C-2_D_:Eu*** | -* | 1.15/1.60 | -* | 0.017/0.024 |
| **C-2_E_:Eu** | 1.12(3)/2.21(7) | 3.07/3.76 | 0.016/0.033 | 0.046/0.057 |

* The significance test did not allow unambiguous determination of the Eu content the case of BiO-NC **C-2_D_:Eu** (for details refer to Table 10, Table 11).

## PXRD

Figure S 24. Powder X-ray diffraction patterns of BiO-NC at room temperature in comparison to the calculated patterns generated from the respective single crystal X-ray diffraction data SC (*T* = 100 K) of **(a)** **C-1_D_:Eu**, **(b)** **C-2_D_:Eu** in mother liquor measured in capillary, **(c)** **C-2_E_:Eu** in mother liquor measured in capillary (SC XRD, *T* = 100 K). Small differences in the patterns are caused by the different temperatures applied performing the measurements (PXRD: *T* = 298 K, SC XRD: *T* = 100 K) and partial loss of packing solvent during grinding the crystals for PXRD studies.

## UV–vis

Table S 18. Summary of the Eu^3+^ transitions with the respective wavelength in the UV–vis DRS spectra of cluster **C-1_D_:Eu** – **C-2:Eu**.

| **BiO-NC** | ^7^F_0_ → ^5^L_6_ | ^7^F_1_ → ^5^L_6_ | ^7^F_0_ → ^5^D_2_ | ^7^F_0_ → ^5^D_1_ | ^7^F_1_ → ^5^D_1_ | ^7^F_0_ → ^5^D_0_ |
| --- | --- | --- | --- | --- | --- | --- |
| **C-1_D_:Eu** | 394 nm | 401 nm | 464 nm | 526 nm | 533 nm | 579 nm |
| **C-2_D_:Eu** | 394 nm | 401 nm | 466 nm | 526 nm | 535 nm | 580 nm |
| **C-2:Eu** | 394 nm | 401 nm | 465 nm | 526 nm | 535 nm | 580 nm |

## Photophysical studies of undoped and Eu^3+^ doped BiO-NCs

### Photophysical studies of undoped-clusters **C-1_D_** and **C-2**

Figure S 25. Commission Internationale de l’Eclairage (CIE) 1931 coordinates X = 0.3731 and Y = 0.5088 of **C-1** and X = 0.4871 and Y = 0.4763 **C-2**. The clusters were excited at 314 nm at 2.4 K.

Figure S 26. Photoluminescence profiles of undoped BiO-NC **C-1_D_** at 2.4 K.

### Lifetime studies of **C-1_D_** and **C-2** at 2.4 K

Figure S 27. Photoluminescence lifetime decay plot of undoped BiO-NC **C-1_D_** (left) and **C-2** (right) at 2.4 K. The decay is fitted with a biexponential function to extract lifetime, summarized in Table S 19. The sample was excited at 314 nm.

Table S 19. PL lifetimes of the undoped clusters measured at 2.4 K.

| Cluster | Lifetimes (*τ*/μs) | |
| --- | --- | --- |
|  | *τ_1_* | *τ_2_* |
| **C-1_D_** | 65 ± 5 | 143 ± 16 |
| **C-2** | 38 ± 1 | 110 ± 2 |

## Temperature-dependent photophysical studies of the Eu^3+^ doped BiO-NCs

### Photoluminescence excitation (PLE), photoluminescence (PL), and lifetime studies of the Eu^3+^ doped BiO-NCs at 2.4 K

PL and PLE Studies of the Eu^3+^ doped BiO-NCs at 2.4 K

Table S 20. Summary of the most intense Eu^3+^ transitions with the respective wavelength range in the PL excitation spectra exemplarily summarized for cluster **C-1_D_:Eu** at 2.4 K. The values are given in nm.

| **BiO-NC** | ^7^F_0_ → ^5^D_4_ | ^7^F_0_ → ^5^G_4_ | ^7^F_0_ → ^5^L_6_ | ^7^F_1_ → ^5^L_6_ | ^7^F_0_ → ^5^D_2_ | ^7^F_0_ → ^5^D_1_ |
| --- | --- | --- | --- | --- | --- | --- |
| **C-1_D_:Eu** | 356 – 364 | 371 – 380 | 387 – 396 | 396 – 406 | 458 – 468 | 520 – 528 |

Table S 21. Summary of the Eu^3+^ transitions with the respective wavelength range in the PL emission spectra exemplarily summarized for cluster **C-1_D_:Eu** at 2.4 K. The values are given in nm.

| **BiO-NC** | ^5^D_0_ → ^7^F_0_ | ^5^D_0_ → ^7^F_1_ | ^5^D_0_ → ^7^F_2_ | ^5^D_0_ → ^7^F_3_ | ^5^D_0_ → ^7^F_4_ |
| --- | --- | --- | --- | --- | --- |
| **C-1_D_:Eu** | 578 – 582 | 586 – 599 | 604 – 633 | 646 – 670 | 672 – 716 |

Figure S 28. Excitation and emission depended photoluminescence profiles of **C-2_D_:Eu** emission (left) and of **C-2:Eu** emission (right) at 2.4 K.

Figure S 29. Excitation wavelength dependence of **C-2_D_:Eu** emission (left) and of **C-2:Eu** emission (right) at 2.4 K.

### Lifetime studies of the Eu doped clusters at 2.4 K

Figure S 30**.** Photoluminescence lifetime decay plots of **C-1_D_:Eu** at 2.4 K. (a) The sample was excited at 314 nm and the decay is fitted with a biexponential function to extract the lifetime. (b) The sample was excited at 314 nm and the decay is fitted with a biexponential function to extract lifetime. (c) The sample was excited at 524 nm and the decay is fitted with a monoexponential function to extract the lifetime.

Figure S 31. Photoluminescence lifetime decay plots of **C-2_D_:Eu** at 2.4 K. (a) The sample was excited at 330 nm and the decay is fitted with a biexponential function to extract the lifetime. (b) The sample was excited at 330 nm and the decay is fitted with a biexponential function to extract the lifetime. (c) The sample was excited at 524 nm and the decay is fitted with a monoexponential function to extract the lifetime.

Figure S 32. Photoluminescence lifetime decay plots of **C-2:Eu** at 2.4 K. (a) The sample was excited at 326 nm and the decay is fitted with a monoexponential function to extract the lifetime. (b) The sample was excited at 326 nm and the decay is fitted with a biexponential function to extract the lifetime. (c) The sample was excited at 524 nm and the decay is fitted with a monoexponential function to extract the lifetime.

### Compilation of the PLE and PL profiles of the Eu^3+^ doped BiO-NCs ranging from 2.4 K to 300 K

Figure S 33. Temperature-dependent PLE and PL studies of (a) **C-1_D_:Eu**, (b) **C-2_D_:Eu**, (c) **C-2:Eu**.

### Photoluminescence excitation (PLE), photoluminescence (PL), and lifetime studies of the Eu^3+^ doped BiO-NCs at 77 K

Figure S 34. Excitation and emission depended photoluminescence profiles of (a) **C-1_D_:Eu**, (b) **C-2_D_:Eu** and (c) **C-2:Eu** emission at 77 K.

Figure S 35. Excitation wavelength dependence of (a) **C-1_D_:Eu**, (b) **C-2_D_:Eu** and (c) **C-2:Eu** emission at 77 K.

Figure S 36. Photoluminescence lifetime decay plots of **C-1_D_:Eu** at 77 K, (a) was excited at 314 nm and (b) was excited at 524 nm. The decay is fitted with a monoexponential function.

Figure S 37. Photoluminescence lifetime decay plots of **C-2_D_:Eu** at 77 K, (a) was excited at 330 nm and (b) was excited at 524 nm. The decay is with a monoexponential function.

Figure S 38. Photoluminescence lifetime decay plots of **C-2:Eu** at 77 K, (a) was excited at 326 nm and (b) was excited at 524 nm. The decay is fitted with a monoexponential function.

### Photoluminescence excitation (PLE), photoluminescence (PL), and lifetime studies of the Eu^3+^ doped BiO-NCs at 180 K

Figure S 39. Excitation and emission depended photoluminescence profiles of (a) **C-1_D_:Eu**, (b) **C-2_D_:Eu** and (c) **C-2:Eu** emission at 180 K.

Figure S 40. Excitation wavelength dependence of (a) **C-1_D_:Eu**, (b) **C-2_D_:Eu** and (c) **C-2:Eu** emission at 180 K.

Figure S 41. Photoluminescence lifetime decay plot of **C-1_D_:Eu** at 180 K. (a) was excited at 314 nm and (b) was excited at 524 nm. The decay is fitted with a monoexponential function.

Figure S 42. Photoluminescence lifetime decay plot of **C-2_D_:Eu** at 180 K. (a) was excited at 330 nm and (b) was excited at 524 nm. The decay is fitted with a monoexponential function.

Figure S 43. Photoluminescence lifetime decay plot of **C-2:Eu** at 180 K. (a) was excited at 326 nm and (b) was excited at 524 nm. The decay is fitted with a monoexponential function.

### Photoluminescence excitation (PLE), photoluminescence (PL), and lifetime studies of the Eu^3+^ doped BiO-NCs at 300 K

Figure S 44. Excitation and emission depended photoluminescence profiles of (a) **C-1_D_:Eu**, (b) **C-2_D_:Eu** and (c) **C-2:Eu** emission at 300 K.

Figure S 45. Excitation wavelength dependence of (a) **C-1_D_:Eu**, (b) **C-2_D_:Eu** and (c) **C-2:Eu** emission. The plots in blue represent ^5^D_0_ → ^7^F_0_ emission arising from excitation of ^5^D_3_ ← ^7^F_0_ (415 nm) and ^5^D_1_←^7^F_0_ (533 nm ‒ 534 nm) transitions corresponding to Eu^3+^.

Figure S 46. Photoluminescence lifetime decay plot of **C-1_D_:Eu** at 300 K. (a) was excited at 314 nm and (b) was excited at 524 nm. The decay is fitted with a monoexponential function.

Figure S 47. Photoluminescence lifetime decay plot of **C-2_D_:Eu** at 300 K. (a) was excited at 337 nm and (b) was excited at 524 nm. The decay is fitted with a monoexponential function.

Figure S 48. Photoluminescence lifetime decay plot of **C-2:Eu** at 300 K. (a) was excited at 326 nm and (b) was excited at 524 nm. The decay is fitted with a monoexponential function to extract the lifetime.

Table S 22. Temperature-dependent lifetimes of **C-1_D_:Eu**. The emission was monitored at 611 nm; all lifetimes are in μs.

| Temperature (K) | Excitation wavelength (nm) | |
| --- | --- | --- |
|  | 314 | 524 |
| 2.4 | 97± 1 and 1720 ± 10 | 1898 ± 10 |
| 77 | 1797 ± 16 | 1866± 10 |
| 180 | 1714 ± 4 | 1873 ± 12 |
| 300 | 1598 ± 6 | 1636 ± 12 |

Table S 23. Temperature-dependent lifetimes of **C-2_D_:Eu**. The emission was monitored at 611 nm; all lifetimes are in μs.

| Temperature (K) | Excitation wavelength (nm) | |
| --- | --- | --- |
|  | 337 | 524 |
| 2.4 | 85 ± 1 and 1605 ± 9 | 1760 ± 15 |
| 77 | 1502 ± 6 | 1769 ± 31 |
| 180 | 1497 ± 8 | 1620 ± 25 |
| 300 | 1564 ± 4 | 1699 ± 19 |

Table S 24. Temperature-dependent lifetimes of **C-2:Eu**. The emission was monitored at 611 nm; all lifetimes are in μs.

| Temperature (K) | Excitation wavelength (nm) | |
| --- | --- | --- |
|  | 326 | 524 |
| 2.4 | 57 ± 1 and 1706 ± 7 | 1767 ± 9 |
| 77 | 1767 ± 8 | 1863 ± 8 |
| 180 | 1837 ± 9 | 1840 ± 8 |
| 300 | 1740 ± 4 | 1754 ± 10 |

### CIE coordinates of Eu^3+^ doped BiO-NCs at 2.4 K and 300 K

Figure S 49. Temperature-dependent CIE coordinates (x, y) of (a) **C-1_D_:Eu** under excitation at 314 nm at 2.4 K and 300 K, (b) **C-2_D_:Eu** under excitation at 330 nm at 2.4 K and 300 K and (c) **C-2:Eu** under excitation at 330 nm at 2.4 K and 300 K.

### Compilation of the PLE profiles of the Eu^3+^ doped BiO-NCs ranging from 2.4 K to 300 K

Figure S 50. Temperature-dependent PLE characteristics of (a) **C-2_D_:Eu** and (b) **C-2:Eu** excitation. The blue dotted lines indicate the emergence of the ^7^F_1_ → ^5^D_1_ (533 nm) and ^7^F_1_ → ^5^D_3_ (415 nm) transitions at 180 K and 300 K.

Details on the emission profile analysis:

Parameters such as branching ratio (*β_R_*), asymmetry ratio (R), and Judd-Ofelt parameters — *Ω2*, *Ω4*, and *Ω6* — can be used to deduce the coordination environment around Eu^3+^. The intensities of ^5^D_0_ → ^7^F*_J_* (*J* = 0 – 4) transitions can be quantified by determining the branching ratios. The *β_R_* is the ratio between intensity of a particular ^5^D_0_ → ^7^F*_J_* transition (I(^5^D_0_ → ^7^F*_J_*)) and total intensity involving all the ^5^D_0_ → ^7^F*_J_* transitions (∑I(^5^D_0_ → ^7^F*_J_*), as shown in equation 1.

*β_R_* = I(^5^D_0_ → ^7^F*_J_*)/∑I(^5^D_0_ → ^7^F*_J_*)	Eq. 1

The asymmetric ratio (R) reflects on the symmetry of the local coordination environment around Eu^3+^. The electric dipole (ED)-induced ^5^D_0_ → ^7^F_2_ transition is highly sensitive to local coordination environment around the Eu^3+^, whereas the magnetic dipole (MD)-induced ^5^D_0_ → ^7^F_1_ transition is barely influenced by the coordination environment. Consequently, the integral ratios (R) of these bands (I(^5^D_0_ → ^7^F_2_) / I(^5^D_0_ → ^7^F_1_)) can be used to draw conclusions about the degree of ligand-field symmetry around Eu^3+^.

The Judd-Ofelt parameter *Ω2* reflects on short range effects such as local coordination environment around Eu^3+^ and degree of covalency in the metal-ligand interaction. On the other hand, the parameters *Ω4* and *Ω6* reflect on the long-range polarizability of the crystalline environment influenced by refractive index, phonon-mediated vibronic coupling, and rigidity of the crystalline network. We have determined the values of the above discussed parameters from the experimental emission spectra using the spectroscopic properties calculation module in the LUMPAC software.^18^ The following regions are used — ^5^D_0_ → ^7^F_0_ (578 nm to 582 nm); ^5^D_0_ → ^7^F_1_ (586 nm to 599 nm); ^5^D_0_ → ^7^F_2_ (604 nm to 633 nm); ^5^D_0_ → ^7^F_3_ (646 nm to 670 nm); and ^5^D_0_ → ^7^F_4_ (672 nm to 716 nm) to extract the areas.

Table S 25. Parameters obtained from the PL studies of europium doped BiO-NCs at 2.4 K and 300 K. The BiO-NCs were excited at 524 nm to avoid the mixing between the Bi^3+^- and Eu^3+^-based emissions.

| Parameter | 2.4 K | | | 300 K | | |
| --- | --- | --- | --- | --- | --- | --- |
|  | **C-1_D_:Eu** | **C-2_D_:Eu** | **C-2:Eu** | **C-1_D_:Eu** | **C-2_D_:Eu** | **C-2:Eu** |
| *β_R_ (^5^D_0_ → ^7^F_0_)* | 3.04 | 3.39 | 3.27 | 3.28 | 3.18 | 3.37 |
| *β_R_ (^5^D_0_ → ^7^F_1_)* | 16.03 | 14.63 | 16.32 | 15.95 | 14.15 | 15.34 |
| *β_R_ (^5^D_0_ → ^7^F_2_)* | 39.15 | 46.19 | 38.30 | 47.05 | 47.60 | 42.41 |
| *β_R_ (^5^D_0_ → ^7^F_3_)* | 3.70 | 4.77 | 5.05 | 3.95 | 5.28 | 5.20 |
| *β_R_ (^5^D_0_ → ^7^F_4_)* | 38.09 | 31.02 | 37.07 | 29.77 | 29.79 | 33.68 |
| *R* | 2.44 | 3.16 | 2.35 | 2.95 | 3.36 | 2.76 |
| *Ω_2_* | 4.30 x 10^-20^ cm^2^ | 5.59 x 10^-20^ cm^2^ | 4.17 x 10^-20^ cm^2^ | 5.23 x 10^-20^ cm^2^ | 5.97x 10^-20^ cm^2^ | 4.91 x 10^-20^ cm^2^ |
| *Ω_4_* | 9.39 x 10^-20^ cm^2^ | 8.39 x 10^-20^ cm^2^ | 8.91 x 10^-20^ cm^2^ | 7.34 x 10^-20^ cm^2^ | 8.36 x 10^-20^ cm^2^ | 8.65 x 10^-20^ cm^2^ |

By taking advantage of the measured ^5^D_0_ lifetime, we have quantified the magnitude of non-radiative decay rate (*k*_nr_) contributing to the Eu^3+^-luminescence quenching. First, the radiative lifetime (*τ_rad_*) of the ^5^D_0_ state is determined employing equation 2.

*τ_rad_* = [*A_MD_* × (*n*)^3^ × *I_tot_*/*I_MD_*]^-1^	Eq. 2

Where, *A_MD_* is the spontaneous emission probability of the ^5^D_0_ → ^7^F_1_ transition in vacuum (14.65 s^-1^) and *n* is the refractive index of the complex (approximated as 1.5); *I_tot_* and *I_MD_* are total integrated emission intensity and intensity of the magnetic-dipole-induced ^5^D_0_ → ^7^F_1_ transition, respectively. By using the calculated *τ_rad_* and experimentally determined *τ_obs_* (Table 2), we have estimated the rates of radiative (*k_r_*) and non-radiative (*k_nr_*) relaxation processes employing equations 3 and 4, respectively. The intrinsic quantum yields (φ_Eu_) of the clusters are estimated using equation 5. Please be aware that uncertainties on the order of ~10% are to be expected.

k_r_ = 1/*τ_rad_*	Eq. 3

k_nr_ = [(1/ *τ*_obs_) – (*k_r_*)]	Eq. 4

φ_Eu_ = τ_obs_/τ_rad_	Eq. 5

Figure S 51. PL emission spectrum under excitation of **C2_E_:Eu** at 314 nm for 20 min.

Figure S 52. PL emission spectra under excitation at 395 nm (black) and at 465 nm (red) of BiO-NC **C-2_E_.Eu** (*c* = 0.0001 M) in EtOH at 300 K.

# 3. Hybrid Material Characterization

### Temperature-dependent steady-state and time-dependent photoluminescence studies of **HM-C-2_E_:Eu**

Figure S 53 Excitation wavelength dependence of **HM-C-2_E_:Eu** emission at 2.4 K.

Figure S 54. Photoluminescence lifetime decay plots of **HM-C-2_E_:Eu** at 2.4 K. The decay is fitted with a monoexponential function to extract the lifetime. The sample was excited at 305 nm.

PL, PLE, and lifetime studies of **HM-C-2_E_:Eu** at 77 K

Figure S 55. Excitation wavelength dependence of **HM-C-2_E_:Eu** emission at 77 K.

Figure S 56. Photoluminescence lifetime decay plot of **HM-C-2_E_:Eu** at 77 K. The decay is fitted with a monoexponential function to extract the lifetime. The sample was excited at 305 nm.

### PL, PLE, and lifetime studies of **HM-C-2_E_:Eu** at 300 K

Figure S 57**.** Excitation and emission of **HM-C-2_E_:Eu** at 300 K.

Figure S 58. Photoluminescence lifetime decay plot of **HM-C-2_E_:Eu** at 300 K. The decay is fitted with a monoexponential function to extract the lifetime. The sample was excited at 305 nm.

Table S 26. Parameters obtained from the PL study of **HM-C-2_E_:Eu** at 300 K. The sample was excited at 305 nm at 300 K.

| Parameter | 300 K |
| --- | --- |
|  | **HM-C-2_E_:Eu** |
| *β_R_ (^5^D_0_ → ^7^F_0_)* | 3.99 |
| *β_R_ (^5^D_0_ → ^7^F_1_)* | 18.37 |
| *β_R_ (^5^D_0_ → ^7^F_2_)* | 48.45 |
| *β_R_ (^5^D_0_ → ^7^F_3_)* | 3.92 |
| *β_R_ (^5^D_0_ → ^7^F_4_)* | 25.28 |
| *R* | 2.64 |
| *Ω_2_* | 4.68 x 10^-20^ cm^2^ |
| *Ω_4_* | 5.40 x 10^-20^ cm^2^ |

Table S 27. Parameters obtained from the PL study of **HM-C-2_E_:Eu** at 300 K. The sample was excited at 305 nm at 300 K.

| Parameter | 300 K |
| --- | --- |
|  | **HM-C-2_E_:Eu** |
| *τ*_rad_ | 2.92 ms |
| *τ*_obs_ | 2.34 ms |
| φ_Eu_ | 68% |
| *k*_r_ | 342.46 s^-1^ |
| *k_n_*_r_ | 135.73 s^-1^ |

Temperature-dependent PL and PLE of **HM-C-2_E_:Eu** at 2.4 K

Figure S 59. Temperature dependence of PLE and PL of **HM-C-2_E_:Eu.**

Table S 28. Temperature-dependent lifetimes of the **HM-C-2_E_:Eu**. All lifetimes are in μs.

| Temperature (K) | Excitation wavelength (nm) |
| --- | --- |
|  | 305 |
| 2.4 | 2761 ± 13 |
| 77 | 2631 ± 14 |
| 300 | 2339 ± 21 |

Figure S 60. Temperature-dependent CIE coordinates (x, y) of **HM-C-2_E_:Eu**. The hybrid was excited at 305 nm at 2.4 K and 300 K.

Figure S 61. PL emission spectrum under excitation of the hybrid material **HM-C2_E_:Eu** at 314 nm for 20 min.

## UV–vis Hybrid Material

Figure S 62. UV–vis reflection spectra of powdered hybrid material containing BiO-NC **C-2_E_:Eu** (**HM-C-2_E_:Eu**).

## IR Hybrid Materials

Figure S 63. ATR-IR spectra of BiO-NC **C-2_E_:Eu** (red), Co-polymer of HM without cluster (**HM**, black) and hybrid material containing **C-2_E_:Eu** (**HM-C-2_E_:Eu**, orange).

## TGA

TGA Analysis of BiO-NC **C-2_E_:Eu** revealed similar decomposition under air flow as the previously reported undoped cluster **C-2_E_**.^4^ In a first weight loss step (1.5 ω%) up to approximately 150 °C the remaining solvent EtOH molecules (3.5 EtOH assuming all ethanol) or adsorbed water are evaporated. Afterwards stepwise degradation of the cluster shell up to a weight of 79 ω% at approximately 430 °C is observed. A mixture of Bi_2_O_3_ and Bi is formed. At higher temperatures Bi is oxidized to Bi_2_O_3_ as demonstrated by the weight increase up to approximately 750 °C.

Figure S 64. TGA of BiO-NC **C-2_E_:Eu.**

Figure S 65. PXRD pattern of TGA residue (900 °C, Air) for BiO-NC **C-2_E_:Eu** matching with structural data of *β*-Bi_2_O_3_ (ICDD 01-077-5341, blue) and *α*-Bi_2_O_3_ (ICDD 00-027-0053, red).

| Label | Composition | m [ω%] (Δ) | M [g/mol] | ΔM [ω%] |
| --- | --- | --- | --- | --- |
| I | [Bi_38_O_45_(C_4_H_5_O_2_)_24_(EtOH)_(3.5)_]:Eu | 100 |  |  |
| II | [Bi_38_O_45_(C_4_H_5_O_2_)_24_]:Eu | 98.5 | 10617.76 | -1.5 % |
| III | [Bi_38_O_45_]:Eu = 15 Bi_2_O_3_:Eu + 8 Bi^0^ | 78.6 (19.9) | 8661.21 | -18.4 % |
| IV | 19 Bi_2_O_3_:Eu | 81.2 (2.6) | 8853.20 | +1.8 % |

The TGA of the respective hybrid material containing cluster **C-2_E_:Eu** (**HM-C-2_E_:Eu**) in air is dominated by the degradation of the polymer material. This process takes place in three steps, in the first step up to approximately 200 °C the evaporation of solvent and unreacted volatile educts takes place. In the second step, between 240 °Cup to 350 °C (55 ω%), weaker bonds (head–head or tail–tail) and end groups are decomposed, while in the following third step, up to 430 °C (18.5 ω%), random scission, depolymerization, and oxidation take place.^19^ In a last step between 430 °C and 480 °C, a mass loss to 7.8 % is observed with a high heat flow, which we attribute to the decomposition of the cluster to Bi_2_O_3_. The corresponding **HM** without cluster shows only one decomposition step with an onset at 190 °C, which is about 45 K lower than in **HM-C-2_E_:Eu**. The thermal decomposition is completed at a temperature of 389 °C for the **HM**, whereas the process is completed at 466 °C in the **HM-C-2_E_:Eu**, thus demonstrating the increased thermal stability with about 75 K of the **HM-C-2_E_:Eu** due to cross-linking by the cluster.

TGA experiments performed in a nitrogen atmosphere show slightly different degradation characteristics. The hybrid material (**HM**) without the cluster shows a decomposition in three steps. The first two steps between 150 °C and 330 °C are a result of decomposition by vinylidene end groups and weaker bonds (head–head or tail–tail). The decomposition is shifted to lower temperatures due to the lack of oxygen, which stabilizes the radicals formed and thus slows down the decomposition process.^20^ The second step above 330 °C is a result of decomposition by random scission and depolymerization. The starting temperature of decomposition in the cluster-containing hybrid material **HM-C-2_E_:Eu** is shifted by about 100 K to 250 °C as a result of the higher cross-linking and stabilization due to the BiO-NCs. The first step is a result of degradation by vinylidene end groups and weaker bonds. The decomposition then shows a slight transition to a second step at about 340 °C where random scission and depolymerization as well as decomposition of the BiO-NCs takes place.

Figure S 66. TGA of copolymer **HM** (black) and copolymer containing BiO-NCs **HM-C-2_E_:Eu** (red) under air (*left*) and under N_2_ (*right*).

Figure S 67. PXRD pattern of TGA residue (900 °C, air) from the hybrid material matching with structural data of *β*-Bi_2_O_3_ (ICDD 01-077-5341, blue).* marks an unmatching reflex.

## DSC

The DSC of **HM-C-2_E_:Eu** in an atmosphere of nitrogen shows two endothermic peaks. The first peak has an onset at about 250 °C and a maximum at 300 °C, which agrees well with the onset of the decomposition vinylidene end groups at 255 °C of the polymer, as was observed in TGA. The second peak with an onset at 340 °C and a maximum at 370 °C is consistent with further decomposition by random scission and depolymerisation as observed in the TGA experiment. At 435 °C there is a change in sign, which results in an exothermic step, that continues until the final temperature of 500 °C is reached. According to TGA the decomposition of the polymer is complete at about this temperature. The copolymer **HM** shows a similar DSC curve, but has an additional endothermic peak with an onset at 140 °C and a maximum at 216 °C. We attribute this to the evaporation and decomposition of trapped monomers in **HM**.^21^ The same process was still observed when using a higher temperature of 150 °C for the polymerisation. The onset of the peak is consistent with the onset of decomposition as observed in the TGA of **HM**. We therefore conclude that **HM-C-2_E_:Eu** has a higher degree of polymerization as result of the higher crosslinking by the BiO-NCs. As a result, an increased thermal stability shifting the onset of the degradation of about 100 K to higher temperatures is observed. The onset of the second endothermic peak, which occurs at 250 °C, is consistent with the second decomposition step in the TGA and is slightly below the onset of the corresponding peak for **HM-C-2_E_:Eu**. The third endothermic peak, which has an onset at 320 °C, starts at significantly lower temperatures compared to **C-2_E_:Eu**, but is consistent with the final decomposition step in the TGA.

Figure S 68. DSC of the copolymer **HM** and the copolymer containing BiO-NCs (**HM-C-2_E_:Eu**).

Figure S 69. FT-IR spectra of **HM** before (a) and after UV irradiation (*λ* = 365 nm) for 6 h (b). FT-IR spectra of **HM-C-2E:Eu** before (c) and after UV irradiation (*λ* = 365 nm) for 6 h (d). The ground copolymer **HM** and **HM-C-2E:Eu** (*m* = 10 mg) were placed on a glass plate, followed by illumination for 6 h using a UV LED module (UV-365, Starlight) with a sample–LED distance of 1 cm, respectively.

# References:

1.	Miersch, L.; Schlesinger, M.; Troff, R. W.; Schalley, C. A.; Rüffer, T.; Lang, H.; Zahn, D.; Mehring, M., Hydrolysis of a Basic Bismuth Nitrate — Formation and Stability of Novel Bismuth Oxido Clusters. *Chem. - Eur. J.* **2011,** *17*, 6985-6990. DOI: 10.1002/chem.201100673

2.	Miersch, L.; Rüffer, T.; Mehring, M., Organic–inorganic hybrid materials starting from the novel nanoscaled bismuth oxido methacrylate cluster [Bi_38_O_45_(OMc)_24_(DMSO)_9_]·2DMSO·7H_2_O. *Chem. Commun.* **2011,** *47* (22), 6353-6355. DOI: 10.1039/c1cc11299f

3.	Morgenstern, A.; Thomas, R.; Sharma, A.; Weber, M.; Selyshchev, O.; Milekhin, I.; Dentel, D.; Gemming, S.; Tegenkamp, C.; Zahn, D. R. T.; Mehring, M.; Salvan, G., Deposition of Nanosized Amino Acid Functionalized Bismuth Oxido Clusters on Gold Surfaces. *Nanomater.* **2022,** *12* (11), 1815-1832. DOI: 10.3390/nano12111815

4.	Weber, M.; Thiele, G.; Dornsiepen, E.; Weimann, D. P.; Schalley, C. A.; Dehnen, S.; Mehring, M., Impact of the Exchange of the Coordinating Solvent Shell in [Bi_38_O_45_(OMc)_24_(dmso)_9_] by Alcohols: Crystal Structure, Gas Phase Stability, and Thermoanalysis. *Z. Anorg. Allg. Chem.* **2018,** *644* (24), 1796-1804. DOI: 10.1002/zaac.201800350

5.	Sattler, D.; Schlesinger, M.; Mehring, M.; Schalley, C. A., Mass Spectrometry and Gas-Phase Chemistry of Bismuth–Oxido Clusters. *ChemPlusChem* **2013,** *78* (9), 1005-1014. DOI: 10.1002/cplu.201300122

6.	Weber, M.; Rüffer, T.; Speck, F.; Göhler, F.; Weimann, D. P.; Schalley, C. A.; Seyller, T.; Lang, H.; Mehring, M., From a Cerium-Doped Polynuclear Bismuth Oxido Cluster to *β*-Bi_2_O_3_:Ce. *Inorg. Chem.* **2020,** *59* (6), 3353-3366. DOI: 10.1021/acs.inorgchem.9b03240

7.	Thomas, R.; Nguyen, T. N. H.; Weber, M.; Rüffer, T.; Göhler, F.; Deka, A.; Pöppl, A.; Seyller, T.; Tegenkamp, C.; Mehring, M., Atomically precise bismuth oxido nanoclusters: cerium doping for optical modification and supramolecular self-assembly on Au(111). *Nanoscale* **2025,** *17*, 18291-18304. DOI: 10.1039/D5NR02346G

8.	Cruickshank, D. W. J., The accuracy of electron-density maps in X-ray analysis with special reference to dibenzyl. *Acta Crystallogr. Sect. B* **1949,** *2* (2), 65-82. DOI: 10.1107/S0365110X49000175

9.	Cordero, B.; Gómez, V.; Platero-Prats, A. E.; Revés, M.; Echeverría, J.; Cremades, E.; Barragán, F.; Alvarez, S., Covalent radii revisited. *Dalton Trans.* **2008,** (21), 2832-2838. DOI: 10.1039/B801115J

10.	Alvarez, S., A cartography of the van der Waals territories. *Dalton Trans.* **2013,** *42* (24), 8617-8636. DOI: 10.1039/C3DT50599E

11.	Hu, S.-Z.; Zhou, Z.-H.; Robertson, B. E., Consistent approaches to van der Waals radii for the metallic elements. *Z. Kristallogr.* **2009,** *224* (8), 375-383. DOI: 10.1524/zkri.2009.1158

12.	Mantina, M.; Chamberlin, A. C.; Valero, R.; Cramer, C. J.; Truhlar, D. G., Consistent van der Waals Radii for the Whole Main Group. *J. Phys. Chem. A* **2009,** *113* (19), 5806-5812. DOI: 10.1021/jp8111556

13.	Jia, D.; Gong, L.; Li, Y.; Cao, S.; Zhao, W.; Hao, L.; Li, S.; Pang, B.; Zhang, C.; Li, S.; Zhang, W.; Chen, T.; Dong, L.; Zhou, B.; Yang, D., {BiW_8_O_30_} Exerts Antitumor Effect by Triggering Pyroptosis and Upregulating Reactive Oxygen Species. *Angew. Chem., Int. Ed.* **2021,** *60* (39), 21449-21456. DOI: 10.1002/anie.202107265

14.	Mehring, M.; Mansfeld, D.; Paalasmaa, S.; Schürmann, M., Polynuclear Bismuth-Oxo Clusters: Insight into the Formation Process of a Metal Oxide. *Chem. Eur. J.* **2006,** *12* (6), 1767-1781. DOI: 10.1002/chem.200500857

15.	Zhao, J.; Li, H.; Li, Y.; Li, C.; Wang, Z.; Chen, L., Rectangle versus Square Oxalate-Connective Tetralanthanide Cluster Anchored in Lacunary Lindqvist Isopolytungstates: Syntheses, Structures, and Properties. *Cryst. Growth Des.* **2014,** *14* (11), 5495-5505. DOI: 10.1021/cg500675g

16.	Yang, X.; Jones, R. A.; Oye, M. M.; Wiester, M.; Lai, R. J., Influence of metal–ligand ratio on benzimidazole based luminescent lanthanide complexes: 3-D network structures and chloride anion binding. *New J. Chem.* **2011,** *35* (2), 310-318. DOI: 10.1039/C0NJ00518E

17.	Anker, A. S.; Christiansen, T. L.; Weber, M.; Schmiele, M.; Brok, E.; Kjær, E. T. S.; Juhás, P.; Thomas, R.; Mehring, M.; Jensen, K. M. Ø., Structural Changes during the Growth of Atomically Precise Metal Oxido Nanoclusters from Combined Pair Distribution Function and Small-Angle X-ray Scattering Analysis. *Angew. Chem., Int. Ed.* **2021,** *60* (37), 20407-20416. DOI: 10.1002/anie.202103641

18.	Dutra, J. D. L.; Bispo, T. D.; Freire, R. O., LUMPAC lanthanide luminescence software: Efficient and user friendly. *J. Comput. Chem.* **2014,** *35* (10), 772-775. DOI: 10.1002/jcc.23542

19.	Nikolaidis, A. K.; Achilias, D. S., Thermal Degradation Kinetics and Viscoelastic Behavior of Poly(Methyl Methacrylate)/Organomodified Montmorillonite Nanocomposites Prepared via In Situ Bulk Radical Polymerization. *Polymers* **2018,** *10* (5), 491. DOI: 10.3390/polym10050491

20.	Peterson, J. D.; Vyazovkin, S.; Wight, C. A., Kinetic Study of Stabilizing Effect of Oxygen on Thermal Degradation of Poly(methyl methacrylate). *J. Phys. Chem. B* **1999,** *103* (38), 8087-8092. DOI: 10.1021/jp991582d

21.	Choudhary, M. S.; Lederer, K., Thermal degradation of copolymers of 2-hydroxyethyl methacrylate and alkyl methacrylates. *Eur. Polym. J.* **1982,** *18* (12), 1021-1027. DOI: 10.1016/0014-3057(82)90200-2
